# The immunopeptidome landscape associated with T cell infiltration, inflammation and immune editing in lung cancer

**DOI:** 10.1038/s43018-023-00548-5

**Published:** 2023-05-01

**Authors:** Anne I. Kraemer, Chloe Chong, Florian Huber, HuiSong Pak, Brian J. Stevenson, Markus Müller, Justine Michaux, Emma Ricart Altimiras, Sylvie Rusakiewicz, Laia Simó-Riudalbas, Evarist Planet, Maciej Wiznerowicz, Julien Dagher, Didier Trono, George Coukos, Stephanie Tissot, Michal Bassani-Sternberg

**Affiliations:** 1 Ludwig Institute for Cancer Research, University of Lausanne, Lausanne, Switzerland; 2 Department of Oncology, Centre hospitalier universitaire vaudois, Lausanne, Switzerland; 3 Agora Cancer Research Centre, Lausanne, Switzerland; 4 SIB Swiss Institute of Bioinformatics, University of Lausanne, Lausanne, Switzerland; 5 Center of Experimental Therapeutics, Department of Oncology, Centre Hospitalier Universitaire Vaudois, Lausanne, Switzerland; 6 École Polytechnique Fédérale de Lausanne, Lausanne, Switzerland; 7 School of Life Sciences, École Polytechnique Fédérale de Lausanne, Lausanne, Switzerland; 8 International Institute for Molecular Oncology, Poznań, Poland; 9 Poznań University of Medical Sciences, Poznań, Poland; 10 Department of Pathology, Centre hospitalier universitaire vaudois, Lausanne, Switzerland

**Keywords:** Tumour immunology, Immunoediting, Non-small-cell lung cancer, Cancer

## Abstract

One key barrier to improving efficacy of personalized cancer immunotherapies that are dependent on the tumor antigenic landscape remains patient stratification. Although patients with CD3^+^CD8^+^ T cell-inflamed tumors typically show better response to immune checkpoint inhibitors, it is still unknown whether the immunopeptidome repertoire presented in highly inflamed and noninflamed tumors is substantially different. We surveyed 61 tumor regions and adjacent nonmalignant lung tissues from 8 patients with lung cancer and performed deep antigen discovery combining immunopeptidomics, genomics, bulk and spatial transcriptomics, and explored the heterogeneous expression and presentation of tumor (neo)antigens. In the present study, we associated diverse immune cell populations with the immunopeptidome and found a relatively higher frequency of predicted neoantigens located within HLA-I presentation hotspots in CD3^+^CD8^+^ T cell-excluded tumors. We associated such neoantigens with immune recognition, supporting their involvement in immune editing. This could have implications for the choice of combination therapies tailored to the patient’s mutanome and immune microenvironment.

## Abstract (web summary)

Bassani-Sternberg and colleagues perform multiregion immunopeptidomics, genomics and spatial transcriptomics in patient lung cancer samples, demonstrating heterogeneity in the immunopeptidome associated with degrees of immune cell infiltration.

## Main

Tumors are composed of heterogeneous populations of nonmalignant and malignant cells with variable genetic and epigenetic characteristics that shape their ability to coexist and coevolve. This evolutionary process diversifies the expression of tumor antigens, the human leukocyte antigen (HLA) presentation of those antigens to cytotoxic T cells and the induction and the duration of effective anti-tumor immunity. In patients with lung cancer, it has been shown that the tumor immune microenvironment (TME) is highly variable between and within patients^1^. Tumors have been grouped into two main subtypes—infiltrated and excluded—according to the magnitude of infiltration of cytotoxic T cells^2–4^. Patients with infiltrated tumors typically respond better to immune checkpoint blockade (ICB) therapy^5^. Never-smoker patients with lung cancer respond poorly to ICB^6^ and the low responsiveness is thought to be associated with low tumor mutational burden (TMB), low neoantigen load and lower expression of programmed cell death-ligand 1 (PD-L1)^7,8^. In addition, high density of tissue-residence memory T cells within non-small-cell lung cancers (NSCLCs) is associated with response to ICB^9^. However, most patients harbor excluded tumors and even patients with a high TMB may not respond^10^. Moreover, it remains unknown whether the repertoire of HLA-bound peptides presented in T cell-infiltrated lung cancer tumors is substantially different from the repertoire presented in excluded tumors, and which immunogenic antigens mediate tumor killing. Certainly, the rational development of more effective immunotherapy treatments targeting tumor antigens in T cell-infiltrated and -excluded tumors would benefit from a more complete understanding of the tumor antigenic landscape.

Immune editing of tumors is a dynamic process and the timing of immune pressure plays an important role in tumor evolution. Chronic tobacco smoking induces immune surveillance, promoting the growth of tumor clones capable of immune evasion early in carcinogenesis^11^. In a therapeutic setting, clonal neoantigens (that is, detectable in all cancer cells) were shown to have been eliminated after ICB treatment in resistant tumors^12^. It is commonly accepted that clonal mutated neoantigens are ideal targets for vaccine or adoptive cell therapies. However, the clonality and heterogeneity of other tumor-specific canonical and noncanonical antigens^13^ that can potentially manifest tumor recognition are largely unknown. Once identified, these new antigens may serve as biomarkers and guide the development of advanced personalized immunotherapy.

To capture the complex interplay between the tumor antigenic landscape and anti-tumor immunity in lung cancer, we integrated genomics, transcriptomics, immunopeptidomics, spatial transcriptomics and multiplexed immunofluorescence (mIF) imaging to investigate the antigenic landscape in tumors with variable degrees of immune infiltration. We surveyed 61 tumor regions and adjacent nonmalignant lung tissues in 8 patients with lung cancer and performed deep antigen discovery combining HLA-I and HLA-II mass spectrometry-based immunopeptidomics, identified tumor antigens and explored their heterogeneous presentation. We associated diverse immune cell populations with the HLA-II immunopeptidome and identified a panel of source proteins, the presentation of which is associated with either CD3^+^CD8^+^ T cell infiltration or inflammation. We found that CD3^+^CD8^+^ T cell-excluded tumors not only have a higher expression, but also a higher presentation efficiency of tumor-associated antigens (TAAs). A significantly higher frequency of predicted neoantigens within HLA-I presentation hotspots was detected in the excluded tumors and nonsmokers compared with T cell-infiltrated tumors or smokers. With an unbiased external resource of validated immunogenic neoantigens, we associated such neoantigens in presentation hotspots with immune recognition, supporting their involvement in immune editing. Our approach could guide the choice of combination therapies tailored to the patient’s mutanome and the TME.

## Results

### Characterization of the antigenic landscape and the TME

**Fig. 1:**
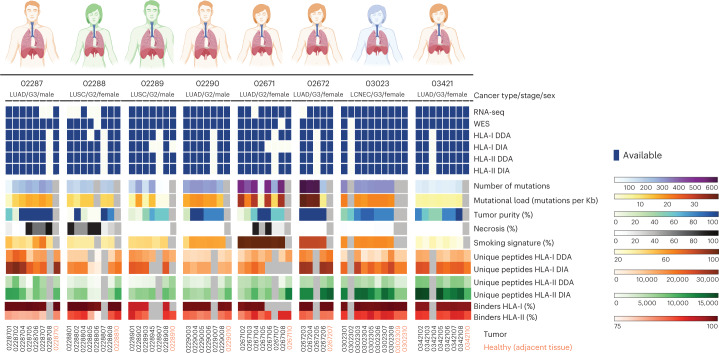
Schematic summary of the lung cancer cohort. A summary of tissues and analyses done on the multiregion tissues, as well as information on the number of somatic mutations affecting protein sequences passing our pipeline’s thresholds, mutational load, tumor purity, necrosis level, number of unique HLA-I and HLA-II peptides identified by mass spectrometry and the percentage of peptides predicted as binders to the respective HLA allotypes (rank <2%). Patient characteristics and processing information can also be found in Supplementary Tables 1 and 2. Source data

**Extended Data Fig. 1:**
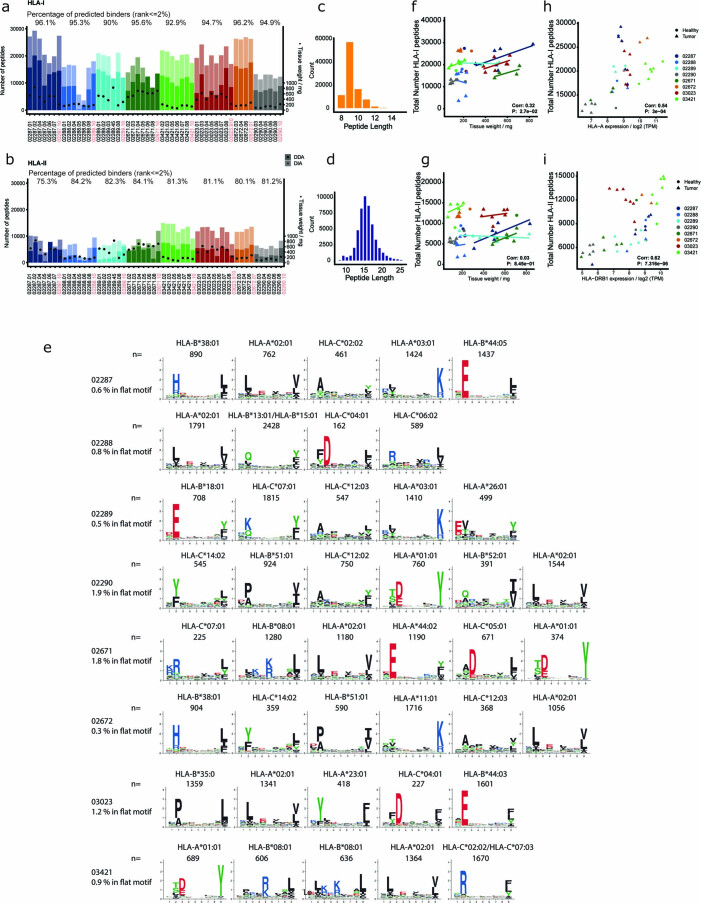
Mass spectrometry based immunopeptidomics performed on the different macro-region tissues. **a**, HLA-I and **b**, HLA-II. DIA (light bars) analyses increased the number of identified peptides by up to 100% compared to DDA (solid bars). Peptide counts in the adjacent healthy macro-regions fall into the same range as the cancer samples. The average percentage of peptides predicted to bind the respective HLA allotypes in each patients are indicated above the bars. Peptide length distributions of **c**, HLA-I and **d**, HLA-II immunopeptidomics datasets. **e** Clustering of randomly selected 5000 HLA-I peptides per patient revealed the expected consensus binding motifs. Multiple specificity was observed for allele HLA-B*08:01 in patient 03421. The number of identified **f**, HLA-I (*n* = 102323 peptides) and **g**, HLA-II bound peptides (*n* = 53343 peptides) correlated with the starting tissue amount per patient (*n* = 53 macro-regions) but not across patients (p values 0.027 and 0.845, respectively). Across patients (N = 8 patients), a positive significant correlation was found between the number of identified **h**, HLA-I and **i**, HLA-II peptides with expression levels of HLA-A (p value 0.0003, Pearson cor= 0.54) and HLA-DRB1 (p value 7.3e-06, Pearson cor=0.62), respectively (*n* = 46 macro-regions with RNAseq and DIA data). For the correlation shown in **f-i** only macro-regions with DIA measurements were included (*n* = 53 macro-regions).

In the present study, we analyzed a collection of multiple lung tumor regions derived from the same masses and paired nonmalignant adjacent lung tissues (here defined as macro-regions) from 8 primary NSCLCs collected in treatment-naive patients. We subjected a total of 61 macro-regions from 5 lung adenocarcinomas (LUADs), 2 lung squamous-cell carcinomas (LUSCs) and 1 large-cell neuroendocrine carcinoma (LCNEC) to deep proteogenomic analyses which included generation of whole-exome sequencing (WES) and bulk RNA-sequencing (RNA-seq) datasets, as well as mass spectrometry-based HLA-I and HLA-II immunopeptidomics, applying data-dependent and -independent acquisition methods (DDA and DIA, respectively)^14^ (Fig. 1 and Supplementary Table 1). We accurately identified, in total, 102,323 HLA-I and 53,343 HLA-II peptides, as corroborated by the high fraction of peptides predicted to bind the respective HLA alleles (ranging from 90% in 02289 to 96.2% in 02672 for HLA-I and from 75.3% in 02287 to 84.2% in 02288 for HLA-II) and the typical peptide length distributions and binding specificities (Fig. 1, Extended Data Fig. 1a–e and Supplementary Tables 2 and 3). The exceptionally low recovery of peptides from samples 02288-5 and 02288-6 was probably due to the highly (95%) necrotic tissue (Fig. 1 and Supplementary Table 1). The number of identified HLA-I and -II peptides correlated with the amount of tissue available for analysis in individual patients (*P* = 0.027) but not across patients (*P* = 0.845; Extended Data Fig. 1f,g). Across patients, the number of HLA-I- and HLA-II-bound peptides correlated with the respective HLA expression as assessed by bulk RNA-seq (*P* = 0.0003 and 7.3 × 10^−6^, respectively; Extended Data Fig. 1h,i), suggesting important interpatient variability. This could relate to variable prevalence of immune cells, which typically express high levels of HLA molecules and may contribute substantially to the measured immunopeptidome.

**Fig. 2:**
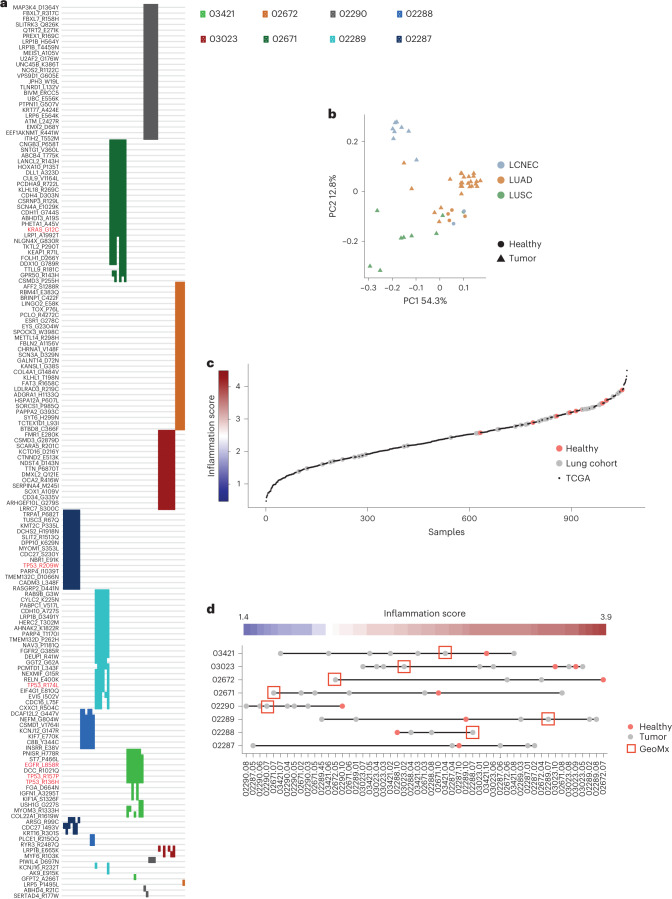
Pathogenic mutations and inflammation scores. **a**, Heat map of detected mutations (*n* = 157 mutations) that were annotated as pathogenic by the FATHMM prediction in COSMIC. Colors represent different patients and every line is a macro-region (*n* = 51 macro-regions). Mutations in *KRAS*, *TP53* and *EGFR* are highlighted in red. **b**, PCA of genes associated with either LUADs or LUSCs confirming the classification of the samples. The list of genes was taken from Reili et al.^15^ and is provided in Supplementary Table 3 (*n* = 53 macro-regions). **c**, Inflammation scores calculated for each macro-region as well as LUAD and LUSC tumors from TCGA using expression levels of the immune-related gene panel as in Danaher et al.^17^. The different macro-regions (*n* = 53 macro-regions) of patients with lung cancer were superimposed on the TCGA data (*n* = 1,011 TCGA patients). **d**, Inflammation scores for each macro-region. The scatter plot denotes 53 regions of the 8 different patients; the red color denotes the healthy samples and red boxes denote the regions subjected to GeoMx analysis. In patient 02287, the tissue selected for GeoMx was not subjected to bulk RNA-seq and therefore not shown in this panel.

As expected, we found pathogenic mutations in oncogenes including *KRAS* and *EGFR* in LUAD samples, and multiple mutations in *TP53* in both LUAD and LUSC samples (Fig. 2a), and prominent smoking mutational signatures were found in patients 02671, 03023, 02672 and 02290 (referred to below as ‘smokers’; Fig. 1). Principal component analysis (PCA) of genes known to be overexpressed exclusively in LUSC or LUAD tumors^15^ confirmed the classification of our samples (Fig. 2b and Supplementary Table 4). We calculated an inflammation score^16^ from bulk RNA-seq data using a defined immune-related gene panel^17^, shown to have optimal performance for lung cancer transcriptomes^1^. We assigned to each macro-region an inflammation status against the landscape of 1,012 LUADs and LUSCs from The Cancer Genome Atlas (TCGA) program (Fig. 2c,d). A wide range of inflammation was observed across patients and within individual patients, whereas the adjacent nonmalignant lung tissues were overall scored as inflamed.

### Spatial analysis of T cell infiltration and inflammation

**Fig. 3:**
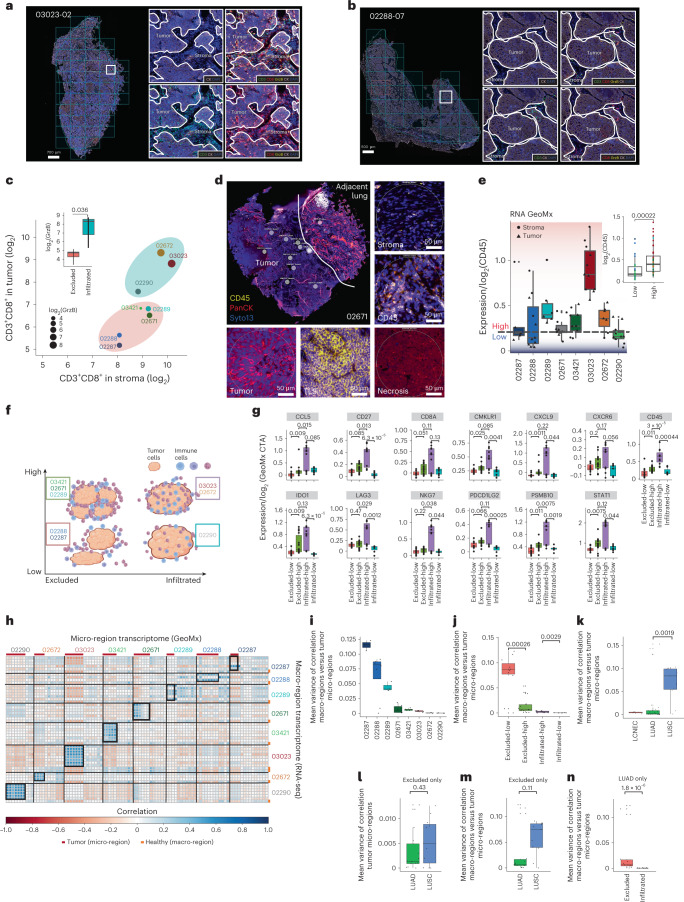
Defining tumors as excluded, infiltrated, immune low and immune high. **a**,**b**, The mIF images of 03023-02 (**a**) and 02288-07 (**b**) demonstrating the masking approach defining infiltration of CD3^+^CD8^+^ double-positive T cells expressing GrzB within tumor and stroma. **c**, The mIF quantification per patient (*n* = 8). Infiltrated samples (*n* = 3) have higher GrzB expression (dot size and inlay plot) and more CD3^+^CD8^+^ T cells in tumor than in stroma (one-sided Student’s *t*-test, *P* = 0.036). **d**, Micro-regions manually selected without independent repetition and classified into tumor, stroma, TLSs, CD45^+^-rich and ‘other’. Five micro-regions of sample 02671, representing 95 micro-regions, are shown. **e**, CD45 expression in tumor and stroma micro-regions calculated from the GeoMx transcriptome. The blue–red line and color scale denote the threshold classifying immune-high and immune-low tumors. Inset: CD45 expression in immune-high (*n* = 44 stroma and tumor micro-regions) or immune-low (*n* = 26 stroma and tumor micro-regions). **f**, Scheme of our relative classification. **g**, Expression in tumor micro-regions of immune activation markers calculated from the GeoMx transcriptome (excluded-high: *n* = 14; excluded-low: *n* = 11; infiltrated-high: *n* = 11; infiltrated-low: *n* = 7). **h**, The transcriptomes of all micro-regions (*n* = 95, GeoMx) were correlated with all macro-regions (*n* = 53, bulk RNA). The black boxes highlight correlations considering tumoral micro-regions per patient. **i**, The mean variance of these correlations in the boxes calculated as variance of correlation coefficients per patient. **j**, Increasing variance from tumors marked as infiltrated-low (02290, *n* = 7 tumor micro-regions), infiltrated-high (03023, 02672, *n* = 11 tumor micro-regions), excluded-high (02289, 02671 and 03421, *n* = 14 tumor micro-regions) and excluded-low (02287, 02288, *n* = 11 tumor micro-regions). **k**, LUSC tumors exhibiting a higher variance. **l**, In excluded tumors, the variance of correlation between tumoral micro-regions shown to be similar in LUADs (02287, 02671 and 03421, *n* = 14 tumor micro-regions) and LUSCs (02288 and 02289, *n* = 11 tumor micro-regions). **m**, The variance of correlation between macro- and micro-regions in excluded tumors. **n**, LUADs showing a significantly higher variance between micro- and macro-regions in excluded tumors (*n* = 14 micro-regions) rather than in infiltrated tumors (*n* = 11 micro-regions). Apart from **c**, one-sided Wilcoxon’s nonparametric tests were used. All boxplots show the median (line), the interquartile range (IQR) between the 25th and 75th percentiles (box) and 1.5× the IQR ± the upper and lower quartiles, respectively. No adjustments were made for multiple testing.

**Extended Data Fig. 2:**
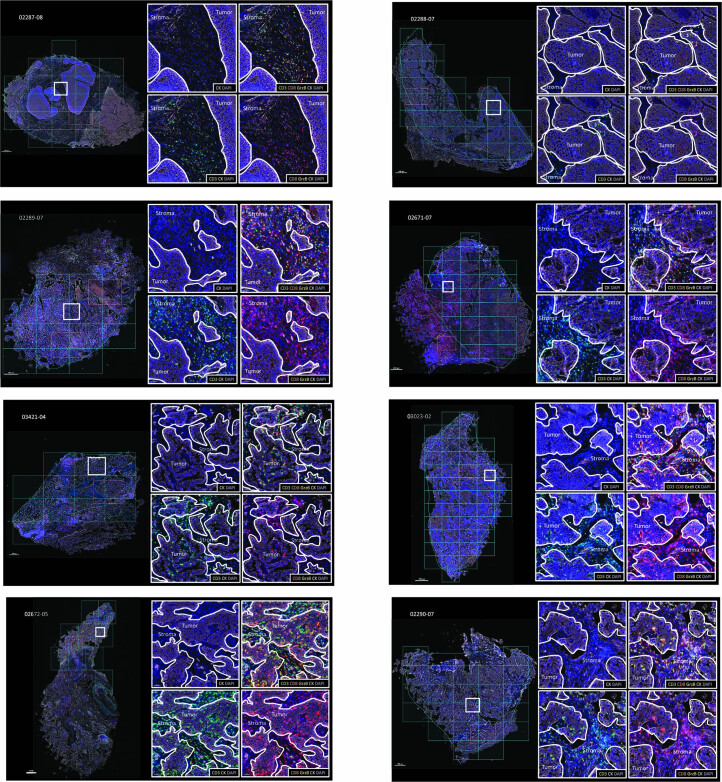
mIF imaging of all patient samples. The masking approach used to define infiltration of CD3^+^CD8^+^ double-positive T cells expressing granzyme B (GrzB) within tumor and stroma niches is shown for all patients.

Immune classification of lung cancer has proven quite challenging. Indeed, immune infiltration, as determined by detailed pathological evaluation, may disagree with infiltration status inferred by gene expression profiles^1^. Therefore, we determined the CD3^+^CD8^+^ T cell infiltration after pathological inspection with hematoxylin and eosin staining and mIF staining of T cell tumor infiltration markers (CD3, CD8, granzyme B (GrzB), Ki67, cytokeratin (CK) and DAPI) (Fig. 3a,b and Extended Data Fig. 2) in one randomly selected macro-region tissue per patient. The level of double-positive CD3^+^CD8^+^ T cells in tumor versus stroma areas and the level of GrzB in the tumor regions were relatively higher in samples 03023, 02290 and 02672. These samples were therefore assigned as CD3^+^CD8^+^ infiltrated and the remaining samples were assigned as CD3^+^CD8^+^ T cell excluded (Student’s *t*-test *P* = 0.036) (Fig. 3c).

The presence of various immune cells is expected to affect the tumor antigenic landscape through potential immune editing, whereas immune cells are expected to contribute directly to the immunopeptidome. To explore the latter, we assessed overall inflammation level (on a scale of high versus low) by spatial transcriptome analyses using the GeoMx Cancer Transcriptome Atlas (CTA) platform. Using CD45, CK and DAPI (to capture immune cells, tumor and epithelial cells, and for segmentation, respectively) we selected for each patient defined micro-regions of interest that were subjected to spatial proteomic and transcriptional analyses. According to the morphological differences and the above markers, the selected micro-regions were annotated as: (1) tumor islets, (2) necrotic, (3) stroma (with variable contributions of tumor cells and immune cells), (4) CD45^+^ (immune) cell rich, (5) tertiary lymphoid structures (TLSs) and (6) other (including blood vessels and nonmalignant lung) (Fig. 3d, Supplementary Fig. 1 and Supplementary Table 5). CD45 expression in tumor and stroma micro-regions was relatively lower in sample 02290 compared with 03023 and 02672, as well as in samples 02287 and 02288 compared with 02289, 02671 and 03421. We therefore assigned samples 02290, 02287 and 02288 as relatively low and the rest as high inflammation (Fig. 3e).

**Extended Data Fig. 3:**
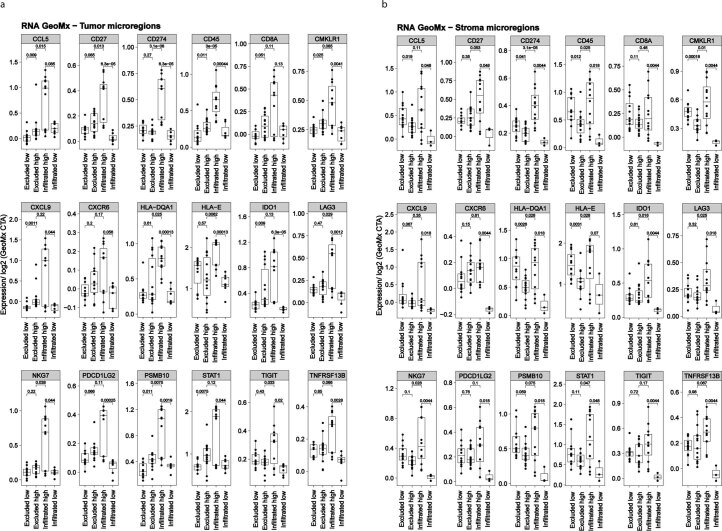
Expression of various immune activation markers calculated from RNA GeoMx transcriptome atlas data. **a**, Expression of immune activation makers in tumor micro-regions (Excluded-high: *n* = 14, excluded-low: *n* = 11, infiltrated-high: *n* = 11, infiltrated-low: *n* = 7 and **b**, stroma Excluded-high: *n* = 15, excluded-low: *n* = 12, infiltrated-high: *n* = 12, infiltrated-low: *n* = 3, *n* refers to tumor micro-regions. Statistical tests have been performed as a one-sided Wilcoxon non-parametric test and, boxplots show the median (line), the interquartile range (IQR) between the 25^th^ and 75^th^ percentile (box) and 1.5*IQR + /- the upper and lower quartile respectively.

Based on the above results, we grouped the patients in a two-dimensional (2D) space relative to each other. On the horizontal axis we ordered the patients on the scale of CD3^+^CD8^+^ T cell infiltration (excluded versus infiltrated) and on the vertical axis based on overall inflammation level (low versus high, Wilcoxon’s test *P* = 0.00022; Fig. 3f). Specifically in tumor micro-regions, the expression of the immune-related genes^18^ *CCL5*, *CD27* (PD-L1), *CD8A*, *CMKLR1*, *CXCL9*, *CXCR6*, *IDO1*, *LAG3*, *NKG7*, *PDCD1LG2* (PD-L2), *PSMB10* and *STAT1* followed the profile of CD45, supporting our classification (Fig. 3g and Extended Data Fig. 3a,b). This rather irregular classification was relevant for downstream assessment of immune editing mediated by CD3^+^CD8^+^ T cells and for the assessment of the global contribution of immune cells to the immunopeptidome. Furthermore, tumoral micro-regions in immune-infiltrated tumors are expected to better ‘mirror’ the bulk tissue because these micro-regions contain components of the immune compartment, as opposed to tumoral micro-regions of immune-excluded tumors. Indeed, correlating the GeoMx gene expression profiles of each tumor micro-region and the respective patient macro-regions’ bulk RNA-seq data revealed increasing variation (calculated as variance of correlation coefficients) from tumors marked as CD3^+^CD8^+^ T cell-infiltrated-low (02290, better mirror), CD3^+^CD8^+^ T cell-infiltrated-high (03023 and 02672), CD3^+^CD8^+^ T cell-excluded-high (02289, 02671 and 03421) and CD3^+^CD8^+^ T cell-excluded-low (02287 and 02288, poor mirror) (Student’s *t*-test *P* = 0.082; Fig. 3h–j), supporting our classification above. It is interesting that, compared with LUADs, LUSC tumors were reported to be more heterogeneous, due to both tumor-intrinsic factors (for example, driver mutations, copy number variations, gene expression profiles) and heterogenic composition of the TME, and these are often linked^19^. Indeed, the above variance of correlations revealed that the two LUSC tumors are more variable than LUADs (*P* = 0.0019; Fig. 3k). We next minimized the bias introduced from the components of the immune compartment by calculating this variance only between tumoral micro-regions in the excluded tumors. The variance in LUAD (02287, 02671 and 03421) and LUSC (02288 and 02289) tumors was similar (*P* = 0.43; Fig. 3l). We then compared the variance of correlation between macro- and micro-regions similarly, only for excluded tumors, and found a higher variation for LUSCs compared with LUADs (*P* = 0.11; Fig. 3m), confirming that these two LUSC tumors are indeed more heterogeneous and the immune compartment may play an important role. Furthermore, considering only the five LUAD cases, we found a significantly higher variance of correlation between micro- and macro-regions in excluded tumors (*P* =1.8 × 10^−6^; Fig. 3n), supporting our conclusion about this complementary approach to validate our classification.

### Biomarkers of immune infiltration in the HLA-II peptidome

**Fig. 4:**
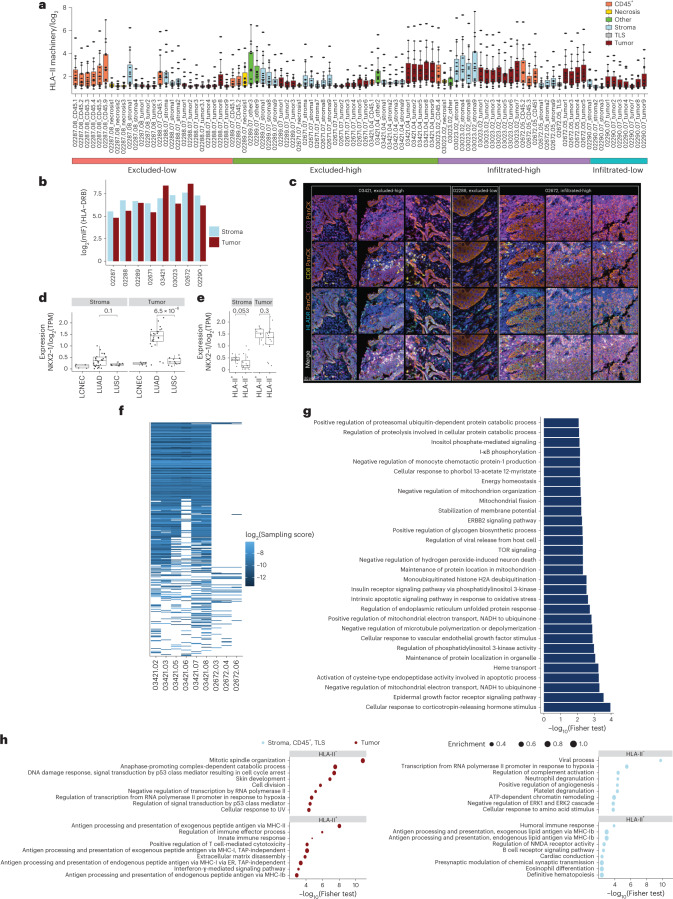
Overview of HLA-II expression. **a**, Expression of genes of the HLA-II presentation machinery (*HLA-DRA*, *HLA-DRB*, *HLA-DRB-3/4/5*, *HLA-DOA*, *HLA-DOB*, *HLA-DQA-1/2*, *HLA-DQB-1/2*, *HLA-DPA1*, *HLA-DPB1*, *HLA-DMA*, *HLA-DMB*, *CTSS* and *CD74*) across all measured GeoMx regions (*n* = 95 micro-regions). **b**, Quantification of *HLA-DRB* expression in stroma and tumor regions by mIF. **c**, HLA-DR molecules expressed on the surface of cancer cells detected only in 03421 and 02672 samples with these tumors assigned as HLA-II^+^, representing *n* = 2 patients. Sample 02288 is shown as an example of an HLA-II^−^ tumor, representing *n* = 6 patients. **d**, Expression of the transcription factor NKX2-1 in stroma (LUADs: *n* = 28; LUSCs: *n* = 9; LCNECs: *n* = 5) and tumor micro-regions (LUADs: *n* = 25; LUSCs: *n* = 11; LCNECs: *n* = 7) in LCNEC, LUAD and LUSC tumors. **e**, Expression of NKX2-1 in stroma, TLS and the CD45^+^ micro-regions (depicted here are stroma) and in tumor micro-regions in HLA-II^+^ (tumor: *n* = 12; stroma: *n* = 16), HLA-II^−^ (tumor: *n* = 16, stroma: *n* = 9) and LUAD tumors. **f**,**g**, HLA-II sampling scores of source genes not found to be presented in any of the healthy tissues and found presented exclusively in HLA-II^+^ tumors (**f**) and their GO enrichment analysis (**g**). TOR, target of rapamycin. **h**, GO analysis of genes with higher expression in HLA-II^+^ (*n* = 12 tumor micro-regions; *n* = 16 stroma, TLS and CD45^+^ micro-regions) versus HLA-II^−^ (*n* = 16 tumor micro-regions; *n* = 19 stroma, TLS and CD45^+^ micro-regions). ER, endoplasmic reticulum; NMDA, *N*-methyl-d-aspartate; UV, uiltraviolet light. Top terms, according to the *P* value (Fisher’s exact test), are displayed. All statistical tests have been performed as one-sided Wilcoxon’s nonparametric test. All boxplots show the median (line), the IQR between the 25th and 75th percentiles (box) and 1.5× the IQR ± the upper and lower quartiles, respectively. No adjustments were made for multiple testing.

**Extended Data Fig. 4:**
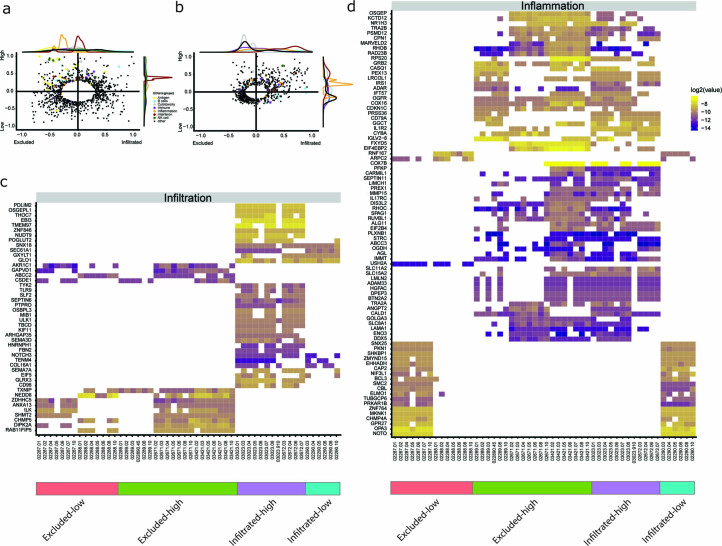
HLA-II peptides as biomarkers of infiltration and inflammation. 2D Gene-Ontology enrichment analysis on **a**, the gene level (bulk RNAseq) and on **b**, the HLA-II presentation sampling score of source genes (HLA-II peptidomics). Immune associated terms were highlighted in color. GO categories with a combined rank (distance to origin) smaller than 0.3 and 0.2 for RNA and DIA respectively are not displayed. HLA-II presented source genes mapped to the GO terms shown in **c**, were filtered further to retain those genes associated with infiltration, which are exclusively present in tumors from **c**, either infiltrated or excluded samples and **d**, those associated with inflammation, that were found exclusively in either immune-high or -low samples. Only source genes detected in ≥50% of the macro-regions were retained.

HLA-II complexes are often abundantly and constitutively expressed on various immune cells in the TME. Furthermore, tumor-intrinsic and -extrinsic factors may influence their expression on the malignant cells. To investigate how such factors influence the HLA-II immunopeptidome, we first assessed the expression of the HLA-II presentation machinery in the different micro-regions. HLA-II machinery expression was higher in infiltrated-high tumor micro-regions compared with other groups, but similar to stroma micro-regions (except sample 03421, as explained below; Fig. 4a). In the CD3^+^CD8^+^ T cell-infiltrated-low sample, the expression of the machinery was higher in tumor micro-regions than in the stroma micro-regions, whereas, in excluded-high and excluded-low samples, the class II machinery was, as expected, more abundant in the stroma than in the tumor micro-regions (Fig. 4a). Next, we constructed a panel of source genes that were exclusively presented along the axis of infiltration (infiltrated versus excluded) and inflammation (high versus low), belonging to enriched immune-related terms (Extended Data Fig. 4 and Supplementary Table 6). For example, toll-like receptor 9 (TLR9) was presented in the HLA-II peptidome of infiltrated samples (03023 and 02672). TLR9 is known to be predominantly expressed by plasmacytoid dendritic cells and B cells^20^ and can reactivate immune surveillance to recognize tumor-specific antigens^21^. These results suggest that the HLA-II peptidome is influenced by the TME and it is a source of biomarkers that capture information about the TME.

To explore this further, we assessed the expression of *HLA-DRB* across tumors and found higher expression in tumor regions than in stroma regions, specifically in the LUAD patients 03421 and 02672 (Fig. 4b), in whom HLA-II molecules were indeed immunolocalized to the membrane of tumor cells (assigned as HLA-II^+^ tumors; Fig. 4c). LUAD predominantly arises from a subset of alveolar type 2 (AT2) cells that are known to constitutively express HLA-II^22,23^. Mouse models suggest that de-differentiation of AT2 cells into a LUAD state is initiated by loss of the lineage transcription factor NKX2-1, which is a master regulator of pulmonary differentiation^24^. NKX2-1 was significantly more abundantly expressed in LUADs compared with LUCSs and LCNECs in tumor micro-regions, and slightly, yet not significantly, more in LUAD HLA-II^+^ tumors (samples 03421 and 02672; Fig. 4d,e). HLA-II peptides derived from source genes that were presented exclusively in the HLA-II^+^ tumors and not in any of the healthy tissues were associated with variable cellular processes (Supplementary Table 7). An interesting example is the category called activation of cysteine-type endopeptidase activity involved in the apoptotic process, including proteins such as CASP4, which is an inflammatory caspase that acts as an essential effector of inflammasomes^25^, and the human growth and transformation-dependent protein (HGTD-P), which promotes intrinsic apoptosis in response to hypoxia^26^ (Fig. 4f,g). HLA-II expression on the LUAD cancer cells may therefore reflect cancer intrinsic and de-differentiation states, but other factors may also be involved. Gene ontology (GO) enrichment analysis of genes overexpressed (*z*-score > 2) in tumor micro-regions of the two above HLA-II^+^ cases (patients 03421 and 02672), relative to all other patients, revealed a significant enrichment for genes associated with processing and presentation of exogenous antigens on HLA-II and on HLA-I, whereas terms related to cell cycle, regulation of transcription and cellular response to DNA damage were mostly enriched in HLA-II^−^ tumors (Fig. 4h); however, these differences were not obvious when stroma, CD45^+^ and TLS micro-regions were analyzed (Fig. 4h). Overall, tumors 03421 and 02672 were classified as CD3^+^CD8^+^ T cell-infiltrated and -excluded tumors, respectively, suggesting a more complex underlying biology associated with the HLA-II immunopeptidome.

### HLA-II peptidome associated with immune cells in the TME

**Fig. 5:**
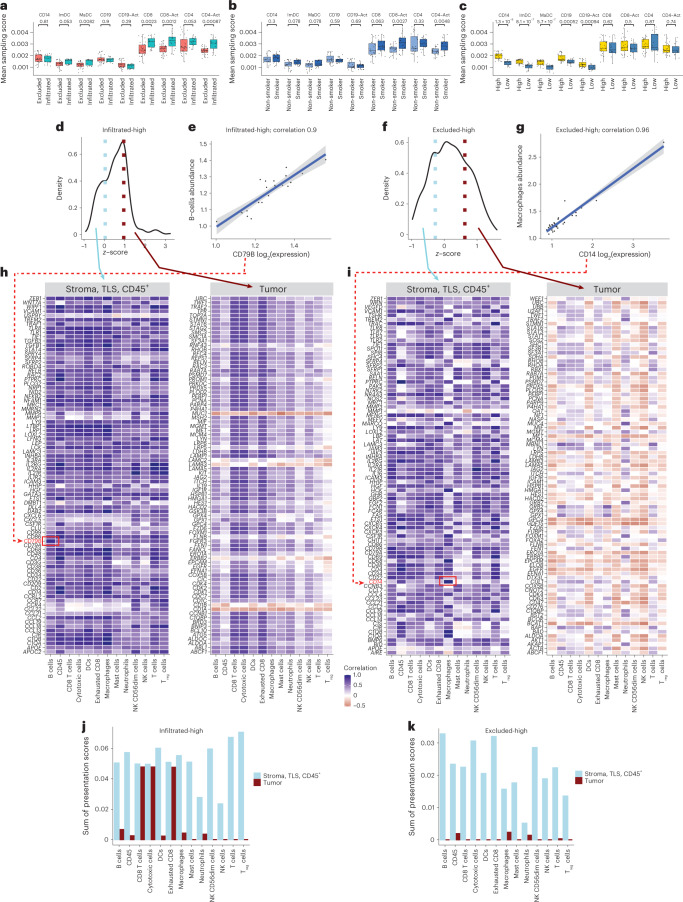
CD3^+^CD8^+^ T cell infiltration impacts the HLA-II immunopeptidome. **a**–**c**, Contribution of immune cells to the HLA-II immunopeptidome based on sampling scores of immune cell markers in tumors annotated as excluded (*n* = 29 tumor macro-regions) (**a**) and infiltrated (*n* = 15 tumor macro-regions), nonsmokers (*n* = 21 tumor macro-regions) and smokers (*n* = 23 tumor macro-regions) (**b**) and immune-high (*n* = 27 tumor macro-regions) and immune-low (*n* = 17 tumor macro-regions) (**c**) per cell type. *P* values were calculated using one-sided Wilcoxon’s test. The boxplots show the median (line), the IQR between the 25th and 75th percentiles (box) and 1.5× the IQR ± the upper and lower quartiles, respectively. No adjustments were made for multiple testing. **d**, The *z*-score distribution of the gene expression comparisons of tumor versus stroma + TLS + CD45^+^ micro-regions in the infiltrated-high samples. Genes in the upper quartile are more highly expressed in tumor micro-regions whereas those in the lower quartile are highly expressed in stroma micro-regions. **e**, Example of correlation of CD79B expression and B cell abundance in infiltrated-high samples (*n* = 26 stroma + TLS + CD45^+^ and tumor micro-regions). The error bands represent the 95% CI. **f**, The *z*-score distribution of the gene expression comparisons of tumor versus stroma + TLS + CD45^+^ micro-regions in excluded-high samples. **g**, Example of correlation of CD14 expression and macrophage abundance in excluded-high tumors (*n* = 34 stroma + TLS + CD45^+^ and tumor micro-regions). The error bands represent the 95% CI. **h**,**i**, Correlation of all genes attributed to stroma + TLS + CD45^+^ micro-regions (lower quartile) or with tumor micro-regions (upper quartile) with cell-type abundance in infiltrated-high (**h**) and excluded-high (**i**) samples. DCs, dendritic cells; NK cells, natural killer cells; T_reg_ cells, regulatory T cells. **j**,**k**, Sum of sampling score for genes correlates with different immune cell type (Pearson’s correlation *r* > 0.5) in infiltrated-high (*n* = 2 patients and *n* = 163 genes) (**j**) and excluded-high (*n* = 3 patients and *n* = 168 genes) (**k**).

**Extended Data Fig. 5:**
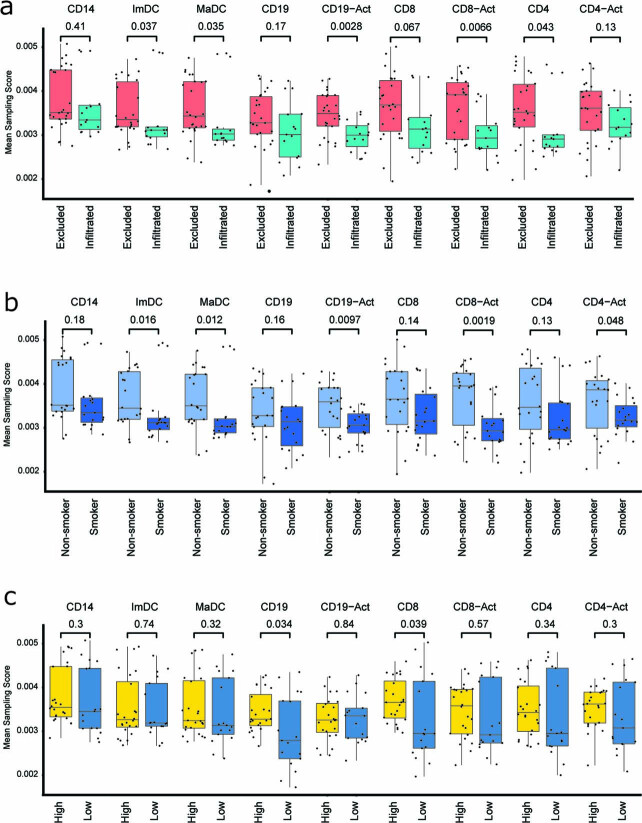
Contribution of immune cells to the HLA-I immunopeptidome. The contribution of immune cells was calculated based on sampling scores of immune cell markers in tumors annotated as **a**, excluded (*n* = 26 macro-regions) and infiltrated (*n* = 15 macro-regions), **b**, non-smokers (*n* = 22 macro-regions) and smokers (*n* = 19 macro-regions), and **c**, immune high (*n* = 24 macro-regions) and low (*n* = 17 macro-regions), per cell type. P values were calculated with a one-sided wilcoxon test. Boxplots show the median (line), the interquartile range (IQR) between the 25^th^ and 75^th^ percentile (box) and 1.5*IQR +/− the upper and lower quartile respectively.

Next, we explored the extent to which immune cell markers are captured by the immunopeptidome in the different groups of tumors. We leveraged a previously published immunopeptidomics dataset of isolated human immune cells before and after in vitro activation, including CD14^+^ precursor cells, immature and mature dendritic cells and CD19^+^ B cells, CD4^+^, CD8^+^ and their corresponding activated cells^27^. For each cell type, we obtained a list of source gene markers that were at >99th and >80th percentiles of the overall sampling score distribution across all the genes, for HLA-I and HLA-II immunopeptidomes, respectively (Supplementary Table 8), and assessed the presentation level of these immune cell markers in our cohort. Remarkably, significantly higher HLA-II presentation levels of CD8^+^ and CD4^+^ T cells, and their activated counterpart cells were found in infiltrated tumors and smokers, but not in the tumors annotated as immune high (Fig. 5a–c). By contrast, CD14^+^, immature and mature dendritic cells, as well as CD19^+^ and activated CD19^+^ cells, were significantly more represented only in the immune-high tumors (Fig. 5a–c). Not surprisingly, the HLA-I immunopeptidome did not reveal as much, potentially because HLA-I molecules are ubiquitously expressed (Extended Data Fig. 5). We concluded that activated CD8^+^ and CD4^+^ T cells are represented in the HLA-II immunopeptidome and even more substantially in their activated states, specifically in tumors annotated as T cell infiltrated and in smokers, whereas the presentation of B cells and dendritic cells is associated with overall high inflammation.

**Extended Data Fig. 6:**
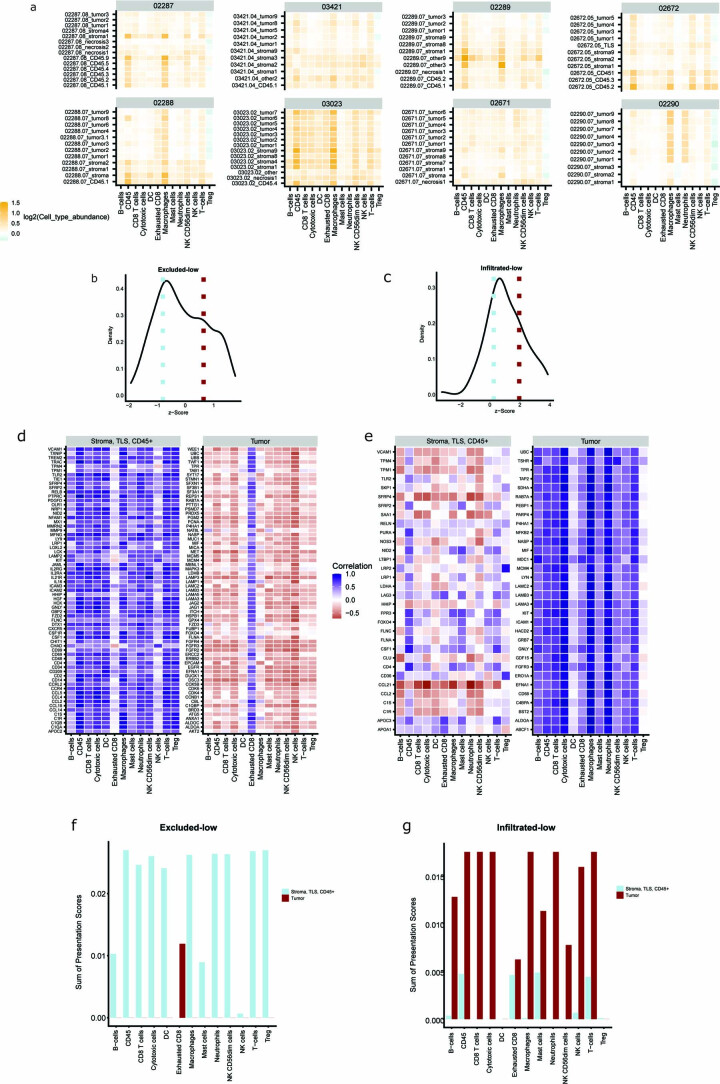
CD3^+^CD8^+^ T cell infiltration impact the HLA-II immunopeptidome. **a**, The relative amount of immune cells in each micro-region calculated on the gene list of Danaher et al. using the GeoMx transcriptome data. Z-score distribution of the gene expression comparisons of tumor versus stroma, TLS, and CD45^+^ micro-regions in **b**, excluded-low (*n* = 2 and 256 genes) and **c**, infiltrated-low (*n* = 1 and 369 genes) samples. Correlation of all genes attributed to stroma, TLS, and CD45^+^ micro-regions (lower quartile) or with tumor micro-regions (upper qurtile) with **d**, cell type abundance in excluded-low and **e**, infiltrated-low samples. Sum of sampling score for genes correlating with any immune cell type (Pearson Correlation r > 0.5) per cell type in **f**, excluded-low (*n* = 2 patients and *n* = 62 genes) and **g**, infiltrated-low samples (*n* = 1 patient and *n* = 169 genes).

With an independent approach guided by the GeoMx transcriptome data, we further explored whether the presence of particular immune cell types in the different micro-regions could affect and contribute to the presented HLA-II immunopeptidome. We calculated the relative amount of immune cells in each micro-region^17^ (Extended Data Fig. 6a). As expected, immune cells were found to be more abundant in the stroma micro-regions than in the tumor micro-regions of excluded-high and excluded-low tumors, and vice versa in the infiltrated-low sample. Next, we focused on all source genes found to be presented in the HLA-II peptidome and further grouped these source genes as tumor related (upper quartile) or stroma, TLS and CD45^+^ related (lower quartile) (Fig. 5d–i), based on their expression in the micro-regions. We correlated their expression with the relative amount of immune cells (Pearson’s correlation coefficient; Fig. 5d–i and Extended Data Fig. 6) in each of the four groups separately. For example, the expression of stroma-, TLS- and CD45^+^-related *CD79B* gene correlated highly with the B cell abundance across all the micro-regions of the T cell-infiltrated-high patient samples (02672 and 03023), and the expression of the stroma-, TLS- and CD45^+^-related CD14 gene correlated highly with macrophages in excluded-high patients (03421, 02289 and 02671) (Fig. 5h,i, respectively). Last, to assess which immune cell types were most associated with the HLA-II peptidome, we summed up, per cell type, the HLA-II presentation sampling scores (which is an approximation of the presentation level) of all genes with Pearson’s correlation coefficient >0.5 (Methods, Fig. 5j,k and Extended Data Fig. 6). It is interesting that the HLA-II peptidome (represented by the presentation of these source genes) of infiltrated-high samples was associated with the presence of CD8^+^ T cells, cytotoxic T cells and exhausted CD8^+^ T cells in the tumor micro-regions, as well as most of the other immune cell types in the stroma, TLS and CD45^+^ micro-regions (Fig. 5j). By contrast, in excluded-high tumors, most of the immune cell types were contributing almost exclusively due to their presence in stroma, TLS and CD45^+^ micro-regions (Fig. 5k). These results highlight the influence that CD3^+^CD8^+^ T cell infiltration has on the HLA-II immunopeptidome.

### HLA-I antigenic landscape and TAA presentation efficiency

**Extended Data Fig. 7:**
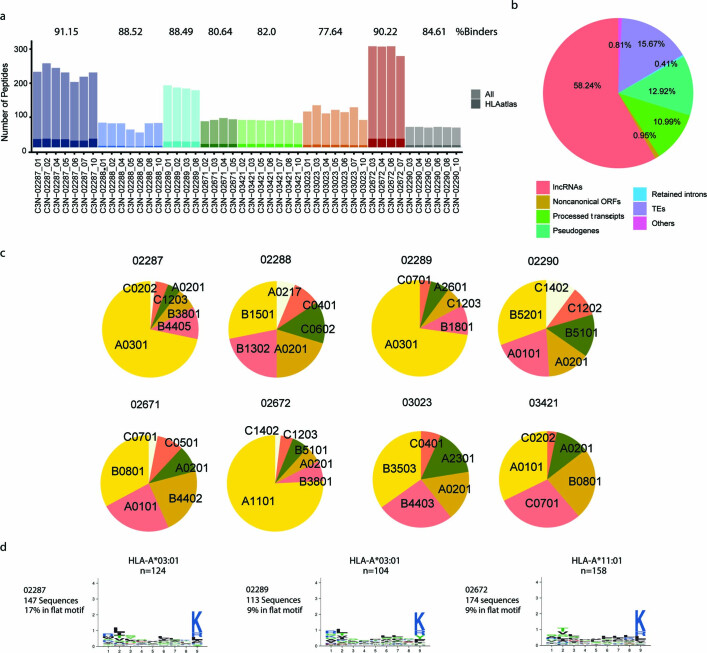
Overview of HLA-I peptides from non-canonical and transposable elements identified by hybrid DIA mass spectrometry based immunopeptidomics. Peptides mapping uniquely to non-canonical and transposable element (TE) sources were analyzed. **a**, numbers of peptides (*n* = 992 peptides) from non-canonical and TE sources, shading indicates peptides also found in the HLA-atlas. **b**, Distribution of identified peptides with respect to gene genomic categories. **c**, Distribution of HLA alleles per patient that have the best binding prediction for all NC/TE peptides (*n* = 992 peptides). **d**, Most important motif for all NC and TE peptides for 3 patients including the percentage of binders and peptide clustering to reveal the binding motifs N is given in the panel.

**Extended Data Fig. 8:**
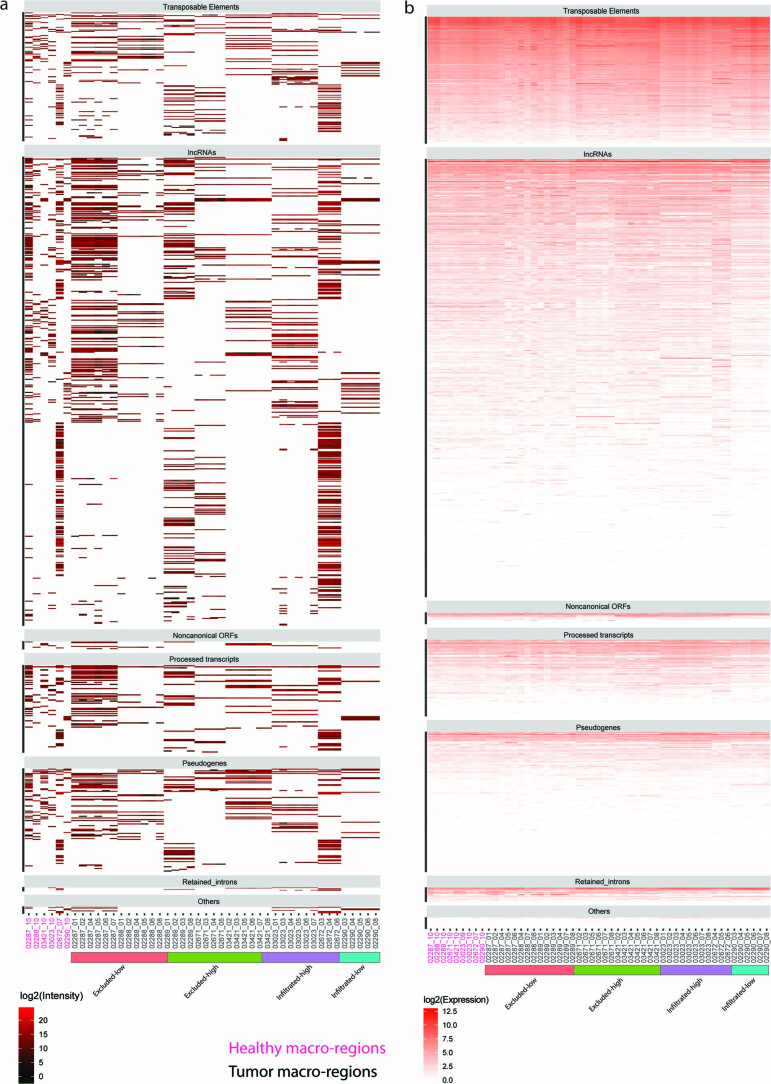
Expression and presentation of non-canonical and TE sources across the macro-regions. Non-canonical and TE sources (*n* = 992 peptides and *n* = 842 source-genes) that were found to be presented were uniformly **a**, presented and **b**, expressed across tumors (*n* = 44 macro-regions) as well as in the adjacent healthy tissues (*n* = 8 macro-regions).

**Extended Data Fig. 9:**
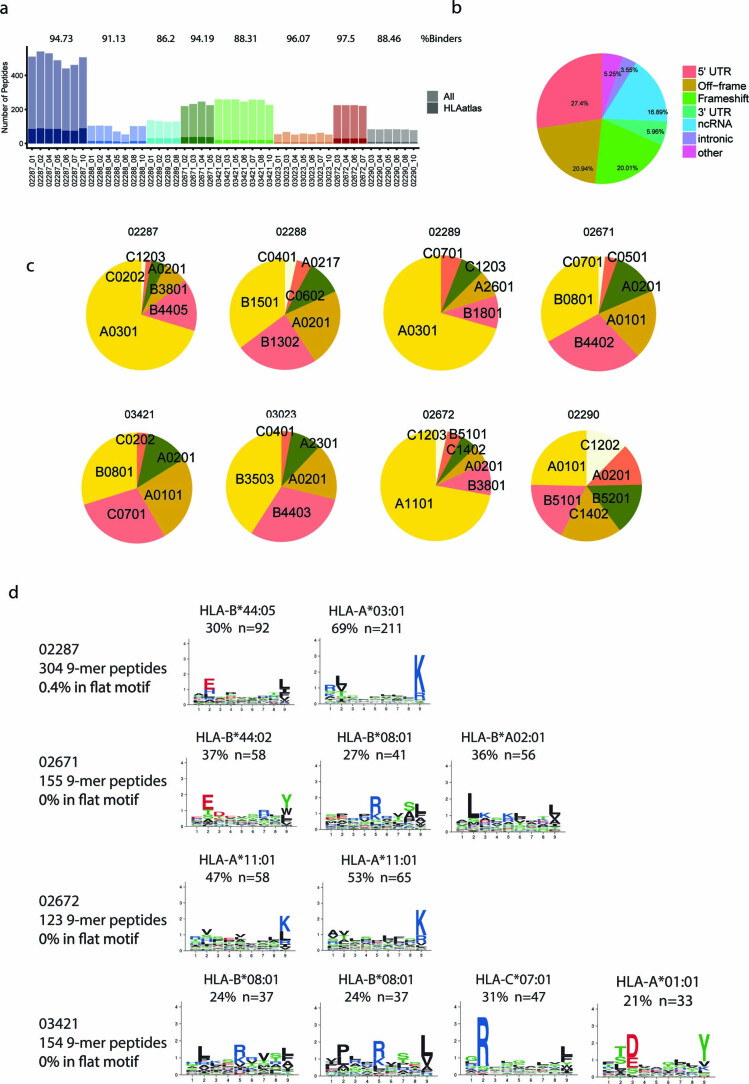
Overview of HLA-I peptides nuORF sources identified by hybrid DIA mass spectrometry based immunopeptidomics. Peptides mapping uniquely to nuORF sources were analyzed **a** numbers of peptides (*n* = 1383 peptides) from nuORF, shading indicates peptides also found in the HLA-atlas. **b** Distribution of identified peptides with respect to seven genomic categories. **c** Distribution of HLA alleles per patient that have the best binding prediction for all nuORF peptides (*n* = 1383 peptides). **d** Most important motifs for all nuORF peptides for four patients. including the percentage of binders and peptide clustering to reveal the binding motifs are shown in the panel.

The global HLA-I peptidome repertoire eluted from bulk tumor tissues is not expected to reveal immune-editing processes because peptides mainly derive from normal proteins and HLA-I molecules are ubiquitously expressed on nontumoral cells. Therefore, we focused on potentially immunogenic source antigens and we matched the mass spectrometry data against customized reference databases that included patient-specific genomic variants (SNPs and somatic mutations), as well as expressed noncanonical genes including long noncoding (lnc)RNAs, transposable elements and a publicly available ribo-seq-derived database of new open reading frames and pseudogenes (nuROFs)^28^ (see Methods for more information and Supplementary Table 9). Although we predicted 812–3,399 HLA-I- and 2,570–10,674 HLA-II-mutated neoantigens (MixMHCpred binding rank ≤2%) across the different samples, we could not detect any by mass spectrometry after manual inspection of tandem mass spectrometry (MS–MS) spectra. Similarly, HLA-II peptides from noncanonical sources were not confidently identified. We identified 18,342 and 12,856 HLA-I and HLA-II peptides, respectively, derived from canonical proteins that were not detected in the immunopeptidomes of adjacent healthy macro-regions and of other benign tissues after re-analysis of the HLA atlas^29^ (Supplementary Tables 2 and 3). Nevertheless, almost all of them were found to be expressed in the adjacent healthy tissues. We detected 218 unique peptides from transposable element sources and 773 unique peptides from other noncanonical sources such as lncRNAs and pseudogenes, but these were uniformly expressed in all tumor macro-regions as well as in the adjacent healthy tissues, indicating no tumor specificity (Extended Data Figs. 7 and 8 and Supplementary Table 9). In addition, most of the 1,409 nuORF-derived peptides were also found presented in the healthy macro-region tissues, with a fraction of those in addition detected in the HLA atlas^29^ (Extended Data Fig. 9 and Supplementary Table 9). The detection of the above noncanonical peptides was associated with HLA allotypes having basic amino acids in the carboxy terminus of their binding motifs, hence, in this small cohort, it was not feasible to associate the presentation level of such a new class of peptides with T cell infiltration.

**Fig. 6:**
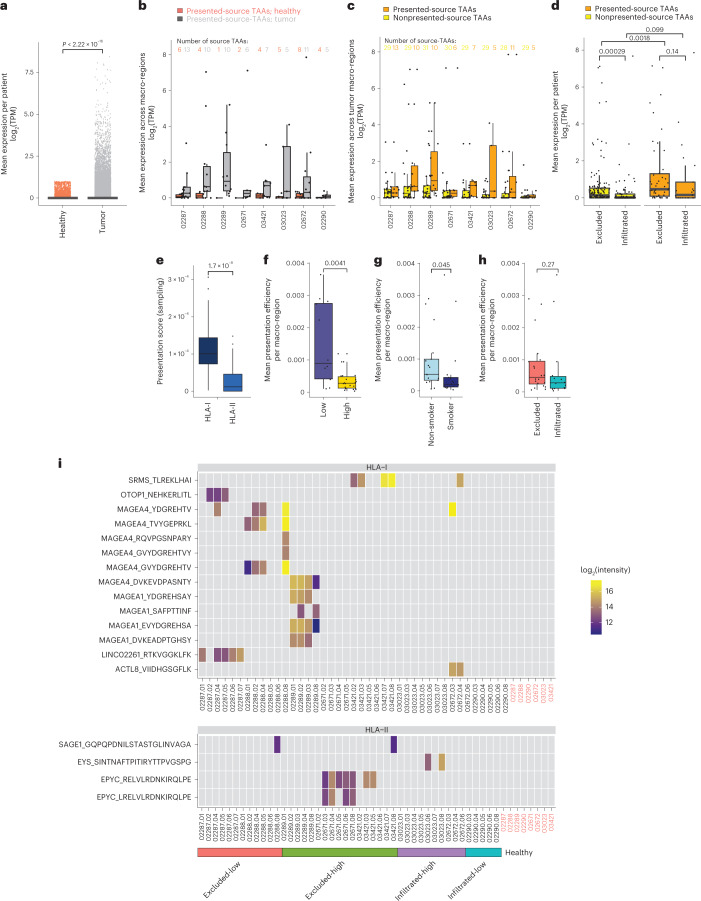
Expression and presentation of tumor-associated genes. **a**, Tumor-associated source genes from canonical and noncanonical sources (*n* = 893 genes), collectively named TAAs, expressed in any of the tumor macro-regions but not in the GTEx databases (GTEx ≤ 1 TPM, except in testis) and not in any of the adjacent healthy macro-regions (≤1 TPM) defined by Wilcoxon’s one-sided test *P* = 2.22 × 10^−16^. No adjustments were made for multiple comparison. **b**,**c**, Across patients, there was higher expression of presented-source TAAs in tumor macro-regions than in the adjacent healthy macro-regions (*n* = 29 TAAs) (**b**) and higher expression of nonpresented-source TAAs (*n* = 31 TAAs) (**c**). **d**,**e**, Presented-source TAAs (*n* = 31 TAAs) expressed more abundantly across CD3^+^CD8^+^ T cell-excluded macro-regions (nonpresented_excluded: *n* = 148; presented_excluded: *n* = 45; nonpresented_infiltrated: *n* = 86; presented_infiltrated: *n* = 22; *n* refers to aggregated TAAs expression per macro-region) (**d**) and presented mainly by HLA-I complexes (averaged across *n* = 41 HLA-I versus *n* = 43 HLA-II macro-regions, respectively*; P* = 1.7 × 10^−8^) (**e**). **f**–**h**, The presentation efficiency of TAAs seen as higher in macro-regions of tumors assigned as immune-low (*n* = 12 macro-regions) versus immune-high (*n* = 22 macro-regions) (**f**), nonsmokers (*n* = 17 macro-regions) versus smokers (*n* = 17 macro-regions) (**g**) and CD3^+^CD8^+^ T cell excluded (*n* = 20 macro-regions) versus infiltrated (*n* = 14 macro-regions) (**h**), with *P* values of 0.0041, 0.045 and 0.27, respectively. **i**, Heat map of source TAAs found to be presented exclusively in tumor macro-regions. Non-normalized log_2_(peptide intensity values) from the DIA analyses are shown. All statistical tests were performed as one-sided Wilcoxon’s nonparametric test.

**Extended Data Fig. 10:**
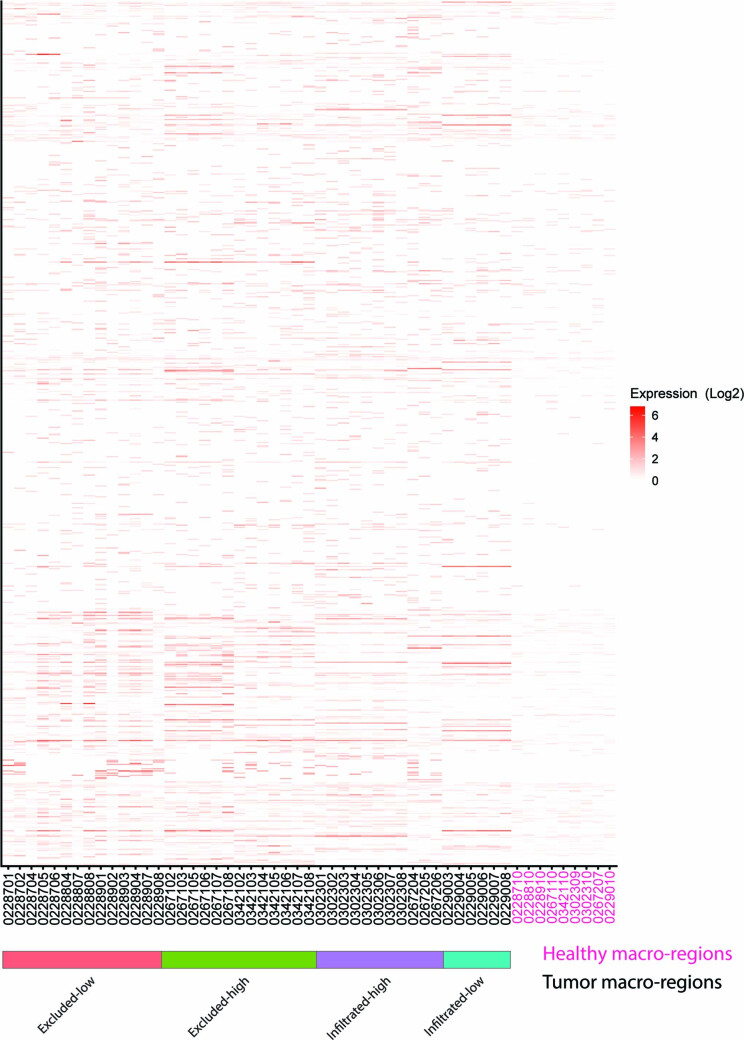
The expression values of the set of tumor specific TAA genes. TAAs were found to be expressed in any of the tumor macro-regions but not in the GTEx databases (GTEx≤1TPM, except in testis) and not in any of the adjacent healthy macro-regions (≤1TPM).

Alternatively, we defined a set of 893 tumor-associated genes derived from canonical and noncanonical sources, collectively named TAAs, which were expressed (>1 transcript per million (TPM)) in at least one tumor macro-region but not in any of the nonmalignant tissues in the Genotype-Tissue Expression (GTEx) database (retaining genes with GTEx expression ≤1 TPM, except in testis) or in any of the adjacent healthy macro-regions (retaining genes with expression ≤1 TPM) (Fig. 6a, Extended Data Fig. 10 and Supplementary Table 10). Of these, 31 source TAAs were found to be presented by HLA-I in at least 1 macro-region in any of the patients. Presented-source TAAs were defined as those detected in the respective macro-region’s HLA-I immunopeptidome, whereas non-presented-source TAAs were those that were not detected, potentially due to lack of presentation resulting from too low expression or limited sensitivity of the immunopeptidomics analyses. Across patients, the expression of presented-source TAAs was higher in tumor macro-regions than in the adjacent healthy macro-regions (Fig. 6b) and higher than the expression of nonpresented-source TAAs (Fig. 6c,d). Furthermore, presented-source TAAs were expressed more abundantly on CD3^+^CD8^+^ T cell-excluded tumors (Fig. 6d) and source TAAs were presented mainly by HLA-I complexes (Wilcoxon’s test *P* = 1.7 × 10^−8^; Fig. 6e). To infer the propensity of a tumor to present TAAs, we computed the mean presentation efficiency of TAAs by normalizing the HLA-I sampling score with TAA gene expression and HLA-I expression levels (Methods). Remarkably, the mean presentation efficiency was higher in macro-regions of tumors classified as immune-low or CD3^+^CD8^+^ T cell excluded, and those of nonsmokers relative to inflamed-high, CD3^+^CD8^+^ T cell-infiltrated samples and smokers (Wilcoxon’s test *P* values of 0.0041, 0.045 and 0.27, respectively) (Fig. 6f–h). This suggests limited immune surveillance that may result in a rather more antigenic immunopeptidome landscape in cohort nonsmokers and CD3^+^CD8^+^ T cell-excluded tumors, and vice versa in smokers and infiltrated tumors.

We further retained source TAAs that were not found to be presented in any of the adjacent healthy macro-regions, resulting in 14 HLA-I and 4 HLA-II peptides (Fig. 6i). Ten HLA-I bound peptides derived from the melanoma-associated gene family sources MAGE-A1 and MAGE-A4, which are known to be expressed in many tumor types but not in normal tissues except for testis and placenta, were expressed and presented mainly in the CD3^+^CD8^+^ T cell-excluded LUSC tumors (that is, 02288 and 02289), supporting a previous study showing an association of MAGE-A4 expression in LUSCs compared with LUADs^30^. MAGE-A4 was the most abundantly expressed and presented TAA, from which six peptides in total were found in four patients and mostly in patient 02288. Furthermore, we found a new tumor-specific, noncanonical peptide in the tumor macro-regions of the CD3^+^CD8^+^ T cell-excluded and nonsmoker patient 02287, derived from the LINC02261 lncRNA.

### Pruning of neoantigens from HLA-I presentation hotspots

**Fig. 7:**
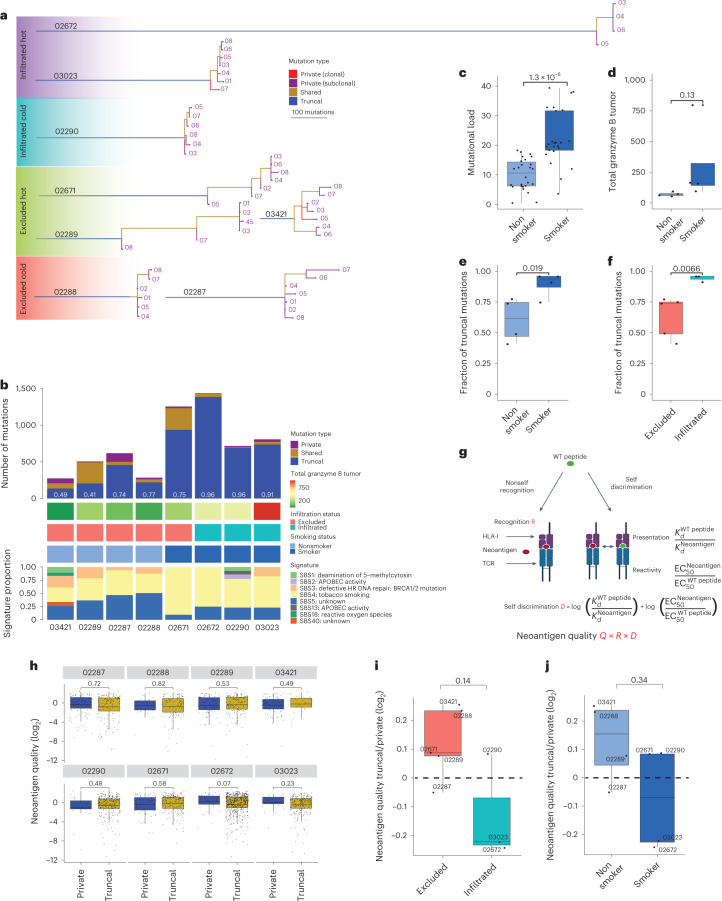
Evidence of neoantigen-mediated immune editing leading to a higher fraction of truncal mutation yet with lower quality. **a**, Phylogenetic trees based on all high-confidence mutations found across all regions per patient. **b**, The number of private, shared and truncal mutations in each patient plotted and fraction of truncal mutations calculated per patient (white numbers). For each patient, GrzB expression in tumor subregions based on mIF analysis and the defined CD3^+^CD8^+^ T cell infiltration status is indicated. Smoking status was defined based on deconvolution of the eight different mutational signatures and comparison to known mutational signatures from Alexandrov et al.^62^ with a threshold of >50% for tobacco smoking signature. **c**,**d**, Positive correlations found between the TMB and the smoking status (smokers *n* = 24 macro-regions; nonsmokers: *n* = 26 macro-regions; one-sided Student’s *t*-test *P* = 1.3 × 10^−6^) (**c**), as well as between the expression of GrzB in tumor subregions (smokers: *n* = 4 patients; nonsmokers: *n* = 4 patients; mIF, one-sided Student’s *t*-test *P* = 0.13) (**d**). **e**,**f**, A higher fraction of truncal (clonal) mutations was found to be significantly associated with smoking status (smokers: *n* = 4 patients; nonsmokers: *n* = 4 patients; one-sided Student’s *t*-test *P* = 0.019) (**e**) and with CD3^+^CD8^+^ T cell infiltration (infiltrated: *n* = 3 patients; excluded: *n* = 5 patients; one-sided Student’s *t*-test *P* = 0.0066) (**f**). **g**, Schematic overview of the predicted neoantigen quality model from Łuksza et al.^32^. **h**, Neoantigen quality score distributions of private and truncal mutations in each patient (02287: *n* = 99/121; 02288: *n* = 26/92; 02289: *n* = 79/130; 03421: *n* = 68/24; 02290: *n* = 21/225; 02671: *n* = 59/187; 02672: *n* = 38 of 489; 03023: *n* = 32/191 (private neoantigens/truncal neoantigens)). **i**,**j**, The ratio between the neoantigen quality of truncal versus private mutations in excluded and infiltrated tumors (excluded: *n* = 5 patients; infiltrated: *n* = 3 patients; boxplot lines show the mean) (**i**), as well as in nonsmokers (*n* = 4 patients) and smokers (*n* = 4 patients) (**j**). Unless indicated otherwise, all statistical tests were performed as one-sided Wilcoxon’s nonparametric test and boxplots show the median (line), the IQR between the 25th and 75th percentiles (box) and 1.5× the IQR ± the upper and lower quartiles, respectively. No adjustments were made for multiple testing.

We defined intratumor heterogeneity by calculating the prevalence of clonal mutations (observed in all macro-regions) and subclonal mutations (observed in a subset of the macro-regions) and inferred each tumor’s phylogeny (Fig. 7a)^31^. We found a positive correlation across TMB, expression of GrzB in tumors and the detection of smoking mutational signatures (Student’s *t*-test *P* values 1.3 × 10^−6^ and 0.13, respectively; Fig. 7b–d). Furthermore, we found that CD3^+^CD8^+^ T cell infiltration (in patients 03023, 02672 and 02290), as well as smoking mutational signatures (in patients 02671, 03023, 02672 and 02290), were significantly associated with higher fractions of truncal mutations (Student’s *t*-test *P* values of 0.0066 and 0.019, respectively; Fig. 7b,e,f). Indeed, Łuksza et al. demonstrated recently that rare long-term pancreatic cancer survivors, who had stronger T cell activity in their primary tumors, developed recurrent tumors with less genetic heterogeneity and fewer high-quality immunogenic neoantigens, despite having more time to accumulate mutations^32^. They modeled neoantigen quality by the antigenic distance required for a neoantigen to differentially bind to the HLA or activate a T cell compared with its wild-type peptide and by the similarity to known antigens (Fig. 7g). In our cohort, we found that the most prominent difference in the quality of neoantigens was found among the truncal and private mutations in the two infiltrated-high patients 03023 and 02672, in whom truncal mutations had lower quality (Fig. 7h–j and Supplementary Table 11). These are evidences of neoantigen-mediated immune editing resulting in truncal tumors in smokers and is consistent with earlier results^33^.

**Fig. 8:**
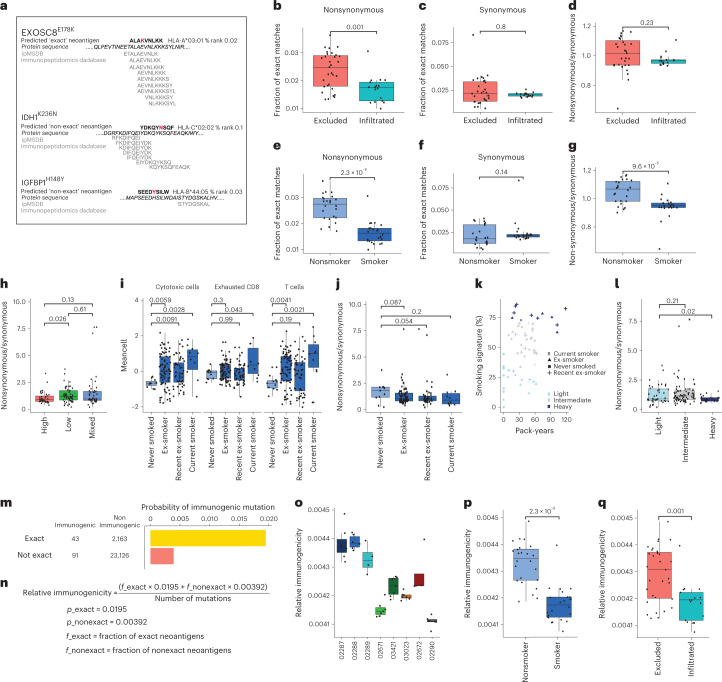
Evidence of neoantigen-mediated immune editing. **a**, EXOSC8^E178K^, an example of ‘exact’ HLA-I presentation hotspot neoantigen. IDH1^K236N^ and IGFBP1^H148Y^ are examples of ‘nonexact’. **b**, The fraction of predicted neoantigens with nonsynonymous mutations matching ‘exact’ wild-type peptides in ipMSDB that is significantly higher in excluded (*n* = 31 macro-regions) than in infiltrated (*n* = 17 macro-regions) tumors (*P* = 0.001). **c**, No difference found when considering predicted neoantigens with synonymous mutations (*P* = 0.8, *n* as in **b**). **d**, Enrichment of ‘exact’ neoantigens in excluded tumors of nonsynonymous versus synonymous mutations per patient (*P* = 0.054). **e**, The fraction of nonsynonymous ‘exact’ neoantigens shown to be significantly higher in nonsmokers (*n* = 24 macro-regions) than in smokers (*n* = 24 macro-regions; two macro-regions were excluded because of lack of neoantigens) (*P* *=* 3.1 × 10^−8^). **f**, No difference found when considering synonymous mutations (*P* = 0.2, *n* as above). **g**, In smokers versus nonsmokers, significant enrichment per patient (*P* = 4.3 × 10^−6^, *n* as above). **h**, Similar enrichment in immune-high (*n* = 38 samples), -low (*n* = 52 samples) and -mixed (*n* = 46 samples) tumors of the TRACERx cohort^1^. **i**, Mean expression of immune markers^17^ in TRACERx cohort grouped by smoking status^1^ (never-smokers: *n* = 11; ex-smokers: *n* = 73; recent ex-smokers: *n* = 48; current smokers: *n* = 10; *n* refers to samples). **j**, The enrichment per smoking status^1^. **k**, TRACERx cohort re-classified (light: *n* = 39; intermediate: *n* = 76; and heavy smokers: *n* = 21; *n* refers to samples), considering mutational signature of tobacco smoking and pack-years^1^. **l**, The enrichment in the refined classification. **m**, Probability of inducing spontaneous CD8^+^ T cell responses to ‘exact’ and ‘nonexact’ neoantigens calculated using Gartner et al.’s cohort of validated immunogenic mutations^35^. **n**, Parameters used to calculate the relative immunogenicity per macro-region. **o**, The relative immunogenicity of our eight patients. **p**,**q**, Relative immunogenicity shown to be higher in nonsmokers (*n* = 24) versus smokers (*n* = 24) (**p**) and in excluded (*n* = 31) versus infiltrated tumors (*n* = 17) (**q**), *P* = 2.3 × 10^−8^ and 0.001, respectively (*n* refers to macro-regions). One-sided Wilcoxon’s nonparametric test was used for **b**–**g**, **p** and **q** and one-sided Student’s **t**-test for **h**–**j** and **l**. Boxplots show the median (line), IQR between the 25th and 75th percentiles (box) and 1.5× the IQR ± the upper and lower quartiles. No multiple testing adjustments were made.

By mining ipMSDB, a large collection of immunopeptidomics databases we acquired in recent years across a variety of tumor and healthy samples, we have previously observed that immunogenic mutated neoantigens accumulate in HLA-I presentation hotspots^34^, that is, regions in source proteins that are more frequently detected in immunopeptidomics datasets. Somatic mutations in these regions are therefore more likely to be presented than mutations in other regions or proteins that are rarely naturally presented. We theorized that, because of the immune-pressure taking place during tumor evolution, cells expressing mutations within HLA-I presentation hotspots will be more frequently eliminated. We predicted in silico HLA-I neoantigen binding to the respective HLA-I allotypes of each patient (rank <2%), and examined for each predicted mutated peptide whether its exact wild-type counterpart peptide was included in the HLA-I presentation hotspot in ipMSDB (Supplementary Table 11). We exemplify this concept in Fig. 8a. The predicted neoantigen covering EXOSC8^E178K^ is an ‘exact’ HLA-I presentation hotspot mutation, whereas the predicted neoantigens IDH1^K236N^ and IGFBP1^H148Y^ do not have a matched ‘exact’ wild-type peptide in ipMSDB. As controls, for each patient we calculated the presence of ‘exact’ matches covering synonymous variants, because these variants are not expected to be affected by immune pressure (Fig. 8a). A higher fraction of ‘exact’ nonsynonymous-predicted neoantigens was found for CD3^+^CD8^+^ T cell-excluded tumors versus infiltrated, whereas no difference was found in the fraction of synonymous mutations (*P* = 0.001 and 0.8, respectively; Fig. 8b,c). We normalized the fraction of nonsynonymous mutations with the fraction of synonymous mutations per patient to eliminate any inherent bias related to the overall representation of the patient’s HLA alleles in ipMSDB. The normalized fractions of ‘exact’ matches almost reached significance (Fig. 8d). A significantly lower fraction of ‘exact’ nonsynonymous-predicted neoantigens was detected also in tumors of smokers (patients 02671, 02290 and 03023, yet not in 02672) relative to nonsmokers (*P* = 2.3 × 10^−8^, Fig. 8e), whereas no difference was found in the fraction of synonymous mutations (*P* = 0.14, Fig. 8f). The normalized fractions of ‘exact’ matches were still significantly lower among smokers (*P* = 9.6 × 10^−5^, Fig. 8g). These results suggest that excessive immune pressure in T cell-infiltrated tumors and smokers may have led to the development of tumors expressing relatively fewer neoantigens within HLA-I presentation hotspots.

To validate these results, we first analyzed samples from 63 patients from the TRACERx lung cancer cohort for which both WES and RNA-seq data were published by Rosenthal et al.^1^ (Methods). Initially, we directly used the immune score classification reported by Rosenthal et al.^1^, who also used the Danaher et al. method^17^ to estimate immune cell populations. With this larger dataset, we again found a higher fraction of ‘exact’ neoantigen matches (enrichment of nonsynonymous/synonymous) in tumors classified as having a low immune score compared with high immune score tumors (Student’s *t*-test *P* = 0.026; Fig. 8h and Supplementary Table 12). Furthermore, as expected, T cells, exhausted CD8^+^ T cells and cytotoxic cells were positively associated with the smoking status documented for these patients (Fig. 8i). Remarkably, a higher enrichment of nonsynonymous/synonymous ‘exact’ matches was observed for never-smokers compared with smokers (Student’s *t*-test *P* = 0.054; Fig. 8j). In addition, when we re-classified the patients into ‘light’, ‘intermediate’ and ‘heavy smokers’, according to the cumulative smoking severity, considering both the level of mutational signature of tobacco smoking and pack-years, we found a significantly higher enrichment of nonsynonymous/synonymous ‘exact’ matches in the ‘light’ group (Student’s *t*-test *P* = 0.02; Fig. 8k,l).

Finally, to assess to what extent predicted mutated neoantigens matching ‘exact’ peptide sequences in ipMSDB can mediate spontaneous CD8^+^ T cell responses in patients, we reanalyzed a large dataset published recently by Gartner et al.^35^, where immunogenicity was assessed by the mini-gene screening approach for thousands of mutations in tens of patients across tumor types. Importantly, this screening method is unbiased because it is not dependent on HLA-binding affinity prediction and, in addition, immunopeptidomics and HLA presentation hotspots information were not considered as selection criteria and therefore could not bias the results. We downloaded data for 77 patients, for which WES, RNA-seq and at least one confirmed immunogenic mutation were available. We analyzed the WES and RNA-seq datasets and flagged the mutations as: ‘immunogenic’, ‘nonimmunogenic’ and ‘not tested’ by the mini-gene approaches (when applicable and as reported by Gartner et al.^35^). We found that mutations predicted to be covered with at least one ‘exact’ match neoantigen have a fivefold higher probability of inducing spontaneous CD8^+^ T cell responses compared with all other mutations (Fig. 8m). We therefore derived the probabilities of a mutation being immunogenic, with *P*_exact_ = 0.0195 and *P*_nonexact_ = 0.00392, and with these probabilities we calculated the relative immunogenicity for each macro-region of our eight patients (see Methods for more details; Fig. 8n). After normalizing for the total number of mutations, the relative immunogenicity of tumors was higher in the nonsmokers than in the smokers, and higher in CD3^+^CD8^+^ T cell-excluded than in CD3^+^CD8^+^ T cell-infiltrated tumors (Student’s *t*-test *P* = 2.3 × 10^−8^ and 0.001, respectively, Fig. 8o–q). These results support our conclusion that ‘exact’ neoantigens are associated with CD3^+^CD8^+^ T cell-mediated recognition and that the lower fraction of ‘exact’ matches in smokers is associated with immune editing.

## Discussion

A key barrier for improving efficacy of advanced personalized immunotherapies that are tailored to specific tumor antigens or the patient’s mutanome, such as neoantigen cancer vaccines and adoptive transfer of neoantigen-enriched T cells, remains patient stratification and the characterization of the antigenic landscape. We therefore aimed to deeply characterize the tumor antigenic landscape and the TME using multiple -omics and imaging approaches. Characterization of the TME from bulk RNA-seq data in lung cancer tissues is challenging, not only in the small cohort we studied here, but also in larger cohorts of tens of samples, as reported by Rosenthal et al.^1^, where lung cancer samples with high inflammation scores were finally classified by pathologists as having low infiltration of cytotoxic T cells and vice versa. Technical variability related to sampling of mirrored formalin-fixed paraffin-embedded (FFPE) tissue sections for staining, and snap-frozen tissues for RNA extraction, which may also include variable amounts of adjacent nonmalignant lung tissue, as well as the natural wide tissue heterogeneity, can be sources of such discrepancies. To overcome this, we applied mIF imaging techniques in combination with GeoMx spatial transcriptome analyses to define niches in the tissues. This approach facilitated the annotation of the samples in a 2D space. On the horizontal axis we ordered the patients on the scale of CD3^+^CD8^+^ T cell infiltration as excluded and infiltrated, and on the vertical axis we ordered them based on overall inflammation level indicative of immune-low and -high tumors. Importantly, mIF and GeoMx data were generated for one macro-region per patient, whereas bulk RNA-seq was done on all macro-regions. However, as the bulk RNA-seq approach was inconsistent for defining the immune compartment using the immunoscore, we did not focus on studying variability between macro-regions of each patient, and instead we compared the groups of patients, considering the different macro-regions as multiple biological replicates per patient.

TAAs were rarely found to be presented by HLA-II complexes. In addition, HLA-II molecules were found to be expressed directly by tumor cells only in samples 03421 and 02672. We therefore hypothesized that the HLA-II peptidome could represent the tumor-immune compartment. Higher or similar gene expression of the HLA-II machinery was found in stroma and tumor micro-regions of T cell-infiltrated samples, whereas in excluded samples, as expected, the machinery was more abundant in the stroma than in the tumor. Activated anti-tumor CD3^+^CD8^+^ T cells secrete interferon-γ that enhances HLA-II expression on neighboring cells in the TME. Hence, insights into the composition of the immune compartment can be uniquely captured by the HLA-II peptidome. We demonstrated that CD8^+^ and CD4^+^ cells were represented in the HLA-II immunopeptidome and even more profoundly in their activated states, specifically in tumors annotated as CD3^+^CD8^+^ T cell infiltrated and in smokers, whereas the presentation of activated B cells and dendritic cells was associated with overall high inflammation. It is interesting that, from the HLA-II presentation level of the source genes that were found to correlate most strongly with different immune cell subtypes in stroma or tumor micro-regions, the presence of CD3^+^CD8^+^ T cells, cytotoxic and exhausted cells in tumor micro-regions distinguished excluded-high and infiltrated-high samples. We have revealed that the HLA-II peptidome was found to capture the presence and activation of immune cells in the TME. Furthermore, we demonstrated associated presentation of several HLA-II peptides with T cell infiltration or inflammation. Therefore, if validated in a larger cohort, the repertoire of HLA-II peptides derived from immune-related genes should allow the classification of a TME. It may help the design of peptide-specific therapeutic modalities by revealing potential tumor-specific targets and reflecting the anti-tumor immune activation state.

So far, it was unclear whether CD3^+^CD8^+^ T cell-excluded tumors express and present TAAs to the same extent as infiltrated tumors. From our results in eight patients with lung cancer, we concluded that, rather unexpectedly, CD3^+^CD8^+^ T cell-excluded tumors express TAAs more abundantly and they have a higher presentation efficiency of TAAs.

Furthermore, we found that the most prominent difference in the quality of neoantigens^32^ was present in infiltrated-high tumors, where truncal mutations had a lower quality. In infiltrated tumors and smokers, mutations were probably edited during tumor evolution^11^. In addition, a significantly higher frequency of predicted neoantigen sequences within HLA-I presentation hotspots was detected in the excluded tumors and in nonsmokers, potentially due to the absence of immune surveillance. This was further validated in the TRACERx cohort. We further demonstrated that the probability to induce spontaneous CD8^+^ T cell responses against mutations predicted to be covered with at least one ‘exact’ match neoantigen was about fivefold higher compared with mutations covered by ‘nonexact’ predicted neoantigens. Accordingly, in our cohort, the relative immunogenicity of tumors was higher in the nonsmokers and CD3^+^CD8^+^ T cell-excluded tumors than in the smokers and T cell-infiltrated tumors, respectively. We therefore propose that accumulation of mutations in presentation hotspots reflects limited immune pressure and lower infiltration of T cells, leading to development of rather heterogeneous and branched tumors.

Nonsmoker patients with lung cancer respond poorly to ICB^6^ and it has been suggested that the low responsiveness is associated with low TMB and lower expression of PD-L1. However, our results from the present study suggest that, even when low in number, neoantigens in nonsmokers and CD3^+^CD8^+^ T cell-excluded tumors have potentially a better chance to be presented to T cells. Consequently, adoptive transfer of neoantigen-enriched autologous T cells, in combination with immune modulators that can revert inhibitory signals in the TME and facilitate homing and persistence of the T cells, could potentially have a therapeutic impact. On the other hand, in CD3^+^CD8^+^ T cell-infiltrated tumors or smokers, too few immunogenic tumor antigens may be presented probably due to prolonged immune editing. In this case, additional therapeutic interventions, for example, epigenetic modulation, targeted therapy, DNA-damaging chemotherapy, irradiation or even hypoxia-inducing anti-angiogenesis therapy, may be needed to induce the expression of new tumor-specific antigens. An integrated exploration of the tumor antigenic landscape and the TME composition would advance the development of personalized immunotherapies that are more effective by tailoring them to clinically relevant tumor antigens for each patient, and identifying which patients are most likely to benefit from these treatments.

## Methods

### Tissue specimens

Lung cancer and adjacent healthy lung tissue samples from eight patients were collected by the International Institute of Molecular Oncology in Poznań, Poland. The different tissue regions were arbitrarily sampled and snap-frozen at −80 °C on surgery.

### HLA typing

High-resolution four-digit HLA-I and HLA-II typing was performed on extracted genomic DNA using the HLA amplification method with the TruSight HLA v.2 Sequencing Panel kit (CareDx). Sequencing was performed on the Illumina MiniSeq System using a paired-end 2× 150-bp protocol. The data were analyzed with Assign TruSight HLA v.2.1 software (CareDx).

### Multispectral immunofluorescence staining

Multiplexed staining was performed on 4-μm FFPE tissue sections on an automated Ventana Discovery Ultra staining module (Ventana, Roche). Detailed information on the antibodies used in each round of multiplex staining is available in the Nature Research Reporting Summary linked to this article.

### Multispectral imaging and data analysis

The mIF images were acquired using the Vectra Polaris, automated, quantitative pathology imaging system (Akoya Biosciences), allowing unmixing of spectrally overlapping fluorophores and tissue autofluorescence of whole-slide scans. For the optimal IF signal unmixing (individual spectral peaks) and the subsequent multiplex analysis, a spectral library containing the individual emitting spectral peaks of all fluorophores was created and validated using the inForm v.2.4.8 Analysis software (Akoya Biosciences). The phenotyping analysis was also performed using inForm. The images were segmented into specific tissue categories of tumor, stroma and no tissue, based on CK and DAPI staining using the inForm Tissue Finder algorithms. Individual cells were segmented using the counterstain-based, adaptive cell, segmentation algorithm. Quantification of the immune cells was performed using the inForm active learning phenotyping algorithm by assigning the different cell phenotypes across several images representing the whole scan. InForm software was trained to recognize cell phenotypes according to the panel. This algorithm was then applied on the selected regions from the whole scan by batch to quantify all the different cell types and an in-house R script was then used to retrieve all combined phenotype cells in an output Excel file. For the analysis, we used cell-type density, which is the above-mentioned abundance per area.

### GeoMx DSP RNA profiling in situ hybridization

Highly multiplexed, spatially resolved profiling experiments were performed with digital optical barcoding technology using the GeoMx Digital Spatial Profiler (DSP) and the CTA (Nanostring) in combination with standard IF according to the manufacturer’s protocol.

Entire slides were imaged at ×20 magnification and morphological markers were used to select the region of interest (ROI) using either circular or organic shapes. ROIs were classified according to CD45 and CK with the supervision of pathologist. Five categories were defined: CD45^+^ (highly enriched in CD45), stroma (CD45^−^ and CK^−^), necrosis (CD45^−^, CK^−^, loss of nuclear staining), TLS (CD45^++^, CK^−^) and tumor (CK^+^, CD45^±^). Then, 95 ROIs were exposed to 385-nm light (ultraviolet), releasing the indexing oligonucleotides, which were collected with a microcapillary and deposited in a 96-well plate for subsequent processing. The indexing oligonucleotides were dried down overnight and resuspended in 10 μl of diethylpyrocarbonate-treated water.

Sequencing libraries were generated by PCR according to the manufacturer’s protocol from the photo-released indexing oligos and ROI-specific Illumina adapter sequences, and unique i5 and i7 sample indices were added. PCR reactions were pooled and purified twice using AMPure XP beads (Beckman Coulter, catalog no. A63881). Pooled libraries were pair sequenced at 2× 27 bp and with the single-index workflow on an Illumina HiSeq 3000/4000 instrument. FastQ files were converted into digital count conversion (DCC) files. DCC files were imported back into the GeoMx DSP instrument for quality control and data analyses using the GeoMx DSP analysis suite v.2.2.0.111. Raw counts were imported into the GeoMx software and adjusted first for technical variability, then scaled by area, and background subtracted, whereby protein targets with a signal:noise ratio <2 were removed. The background probes used were rabbit immunoglobulin (Ig)G, mouse IgG1 and mouse IgG2a. Of 94 regions sampled across patients, only 1 region had <20 nuclei and was automatically excluded from downstream analyses. ROIs were categorized manually based on immunohistochemistry staining and previous knowledge of tumor histology.

### Immunoaffinity purification of HLA peptides

We performed HLA immunoaffinity purification of HLA-I- and HLA-II-bound peptides with W6/32 and HB145 monoclonal antibodies crosslinked to protein A Sepharose 4B (Pro-A) beads according to our previously established protocols^36^. Recovered HLA-I and -II peptides were dried using vacuum centrifugation (Concentrator plus, Eppendorf) and stored at −20 °C. Before mass spectrometry analysis, dried peptides were resuspended in 12 µl of iRT (indexed retention time; Biognosys) peptides diluted 1:10 in 2% acetonitrile and 0.1% formic acid.

### LC–MS/MS analyses

The liquid chromatography–tandem mass spectrometry (LC–MS/MS) system consisted of an Easy-nLC 1200 connected to a Q Exactive HF-X mass spectrometer (Thermo Fisher Scientific). Peptides were separated on a 450-mm analytical column (8-µm tip, 75-µm inner diameter, PicoTipTMEmitter, New Objective) packed with ReproSil-Pur C18 (1.9-µm particles, 120-Å (12-nm) pore size, Dr. Maisch GmbH). The separation was performed at a flow rate of 250 nl min^−1^ by a gradient from 0.1% formic acid to 80% acetonitrile + 0.1% FA.

For DDA, full mass spectrometry spectra were acquired in the Orbitrap from *m*/*z* = 300–1,650 with a resolution of 60,000 (*m*/*z* = 200) and an ion accumulation time of 80 ms. The auto gain control (AGC) was set to 3 × 10^6^ ions. MS/MS spectra were acquired on the 20 most abundant precursor ions with a resolution of 15,000 (*m*/*z* = 200), an ion accumulation time of 120 ms and an isolation window of 1.2 *m*/*z*. The AGC was set to 2 × 10^5^ ions, the dynamic exclusion was set to 20 s and a normalized collision energy (NCE) of 27 was used for fragmentation. No fragmentation was performed for HLA-I peptides with assigned precursor ion charge states of ≥4 or ion charge state of 1 or ≥6 for HLA-II peptides. The peptide match option was disabled.

For DIA, the cycle of acquisition consisted of a full mass spectrometry scan from 300 *m*/*z* to 1,650 *m*/*z* (*R* = 60,000 and ion accumulation time of 60 ms) and 21 DIA MS/MS scans in the Orbitrap. For each DIA MS/MS scan, a resolution of 30,000, an AGC of 3 × 10^6^ and a ramping NCE = 25.5, 27 and 30 were used. The maximum ion accumulation was set to auto and the overlap between consecutive MS/MS scans was 1 *m*/*z*.

### RNA extraction and sequencing

RNA was extracted using the Total RNA Isolation RNeasy Mini Kit with the DNAse I (QIAGEN), on-column digestion step. Snap-frozen pieces of tumor and normal tissue samples (≈30 mg) were directly submerged in 350 µl of RLT buffer (second RNA wash buffer with ethanol) supplemented with 40 µM dithiothreitol. Tissues were completely homogenized on ice using a pestle and passed through a 26G needle syringe 5×. Centrifugation was performed in a table-top centrifuge at 4 °C for 3 min at 18,213*g* before the supernatant was removed and directly used for RNA extraction.

RNA quality was assessed on a Fragment Analyzer (Agilent Technologies). RNA-seq libraries were prepared from 500 ng of total RNA with the Illumina TruSeq Stranded mRNA reagents using a unique dual indexing strategy and following the official protocol automated on a Sciclone liquid handling robot (PerkinElmer). Libraries were quantified by a fluorimetric method (QubIT, Life Technologies) and their quality assessed on a Fragment Analyzer.

Cluster generation was performed with the resulting libraries using Illumina HiSeq 3000/4000 PE Cluster Kit reagents. Libraries were sequenced on the Illumina HiSeq 4000 with HiSeq 3000/4000 SBS Kit reagents for 2× 150 cycles. Sequencing data were de-multiplexed with the bcl2fastq Conversion Software (v.2.20, Illumina).

### DNA extraction and exome sequencing

DNA was extracted with the commercially available DNeasy Blood & Tissue Kit (QIAGEN). Either fresh snap-frozen tissue or pelleted DNA was used. Pelleted DNA was obtained from the pellet collected after centrifugation of the lysed tissue used for the HLA immunoaffinity purification. Pelleted DNA was then resuspended in phosphate-buffered saline using a pestle before DNA extraction.

Genomic DNA (250–500 ng) was fragmented to 150–350 bp using a Covaris S2. Sequencing libraries were then prepared using the KAPA Hyper Prep Library Kit (Roche Sequencing Solutions, Inc.) with xGen UDI-UMI Adapters (Integrated DNA Technologies Inc.). Target enrichment was performed with the Exome research panel v.2 and the xGen reagents according to the manufacturer’s recommendations.

Cluster generation and library sequencing were performed as described above.

### Generation of personalized reference databases

Exome sequence reads were aligned to the Genome Reference Consortium Human Build 37 assembly (GRCh37) with BWA-MEM v.0.7.17 (ref. ^37^). The resulting SAM format was sorted by chromosomal coordinate and converted into a BAM file, then PCR duplicates were flagged, using the Picard AddOrReplaceReadGroups and MarkDuplicates utilities, respectively (from http://broadinstitute.github.io/picard). Quality metrics were assessed using the Picard MarkDuplicates, CollectAlignmentSummaryMetrics and CalculateHsMetrics utilities. GATK BaseRecalibrator (within GATK v.4.1.3.0) was used to recalibrate base quality scores before variant calling^38,39^. The recalibrated tumor and germline BAM files were used as input for ploidy and tumor content estimation by Sequenza (https://pubmed.ncbi.nlm.nih.gov/25319062) and for each of the four variant callers: HaplotypeCaller, MuTect v1, Mutect v2 and VarScan 2 (v.2.4.3). Sequenza was run with default parameters and values of ploidy and tumor content of the model with the highest log(posterior probability score) were selected. HaplotypeCaller^38,39^ was run in genomic variant call format (GVCF) mode on each tumor and germline-recalibrated BAM file to detect SNV and indel (insertion/deletion) variants. The resultant GVCF files were combined using GATK GenotypeGVCF to produce raw variant calls for tumor and germline within a single VCF. Subsequent variant quality score recalibration was performed separately for SNVs and indels using the GATK variant Recalibrator tool to identify high-confidence calls. Variant quality was assessed by the GATK VariantEval tool. Patient-specific SNPs were defined as variants present in both tumor and germline, whereas variants present only in tumor were defined as somatic mutations. The MuTect v.1 variant-calling algorithm was run with default values (--interval_padding 100) and identified somatic mutations were exported in VCF format. The MuTect v.2 variant-calling algorithm was run with default values (--genotype-germline-sites true) and identified variants were exported in VCF format. The multisample pileup file required for VarScan 2 input was generated using SAMtools^40,41^. VarScan 2 was run using default parameters (estimated tumor content from Sequenza was used as --tumor-purity and --min-var-freq was calculated as ${\mathrm{min}}\left( {0.4 \times {\mathrm{estimated}}\,{\mathrm{tumor}}\,{\mathrm{content}},0.2} \right)$) and generated a VCF containing SNVs and indels for both somatic mutations and SNPs. Varscan 2 identified variants were filtered with fpfilter (--dream3-settings).

Variants were combined into a single VCF that contains the union of the variants of all callers. Ambiguous calls were resolved by a simple majority rule, or the call was rejected. GATK WhatsHap v.0.18 (ref. ^42^) was used to retrieve the phasing information of all variants in the combined VCF^38,39^. The functional effect of the variants was annotated by SnpEff. To maximize variant annotation we used annotations from the hg19 (Refseq) and GRCH37.75 (Ensembl) databases^43–45^. From this nonredundant annotated VCF for every macro-region, we created a separate PEFF fasta file for which residue mutation information was added to the header of the affected, translated, protein-coding transcripts^46^.

### RNA-seq analysis and noncanonical sequence database generation

RNA-seq reads were aligned to the GRCH37/hg19 reference genome using RNA-Star (v.2.7.3a; https://github.com/alexdobin/STAR). Raw counts were transformed into TPM values. The comprehensive gene annotation v.32 was downloaded from the GENCODE website (https://www.gencodegenes.org/human/release_32lift37.html) and chromosome position, transcript structure and transcript. and protein sequences were selected to define protein-coding and noncoding genes. For all plots including RNA-seq data we use a log_2_ transformation with a pseudocount of 1. In addition, we mapped RNA-seq reads on transposable elements as previously described^47^. Normalization for sequencing depth was performed for both genes and transposable elements using the trimmed mean of *M* values method with the limma v.3.36.5 package of Bioconductor^48^ and the counts on genes as the library size.

Expressed (TPM > 0.0) noncanonical (lncRNA, polymorphic_pseudogene, processed_pseudogene, pseudogene, TEC, transcribed_processed_pseudogene, transcribed_unitary_pseudogene, transcribed_unprocessed_pseudogene, translated_processed_pseudogene, translated_unprocessed_pseudogene, rRNA_pseudogene, unitary_pseudogene, unprocessed_pseudogene) genomic sequences and the transposable elements were translated in three forward reading frames as identified through a stop-to-stop strategy. Reference sequences, personalized protein-coding sequences and expressed noncanonical and transposable elements entries were merged in a single, sample-specific, personalized proteome.

### MS-based searches

First, for each macro-region, we searched the corresponding raw file against the personalized proteome reference using Comet with precursor mass tolerance 20 p.p.m., MS/MS fragment tolerance of 0.02 Da, peptide length of 8–15 for HLA-I and 8–25 for HLA-II peptides and no fixed modifications, whereas methionine oxidation and phosphorylations on serine, threonine and tyrosine were included as variable modifications. A group-specific, 3% false recovery rate (FDR) for protein-coding, noncanonical sources and transposable elements was calculated by NewAnce v.1.7.1 as previously described^47^. We next generated a single comprehensive reference database containing all the sources of the detected personalized variant and noncanonical peptides from all the patients, and concatenated these to a generic GENCODE database. Then, Comet and NewAnce were run again against this database using the entire cohort immunopeptidomics dataset, and yet separately for HLA-I and HLA-II files, with the same parameters as above. The outputs of this search were used to create spectral libraries for targeted DIA analyses using Spectronaut. The spectral libraries were generated by parsing the PSMs into the BGS generic format by Spectronaut (v.14.6.2, Biognosys). The exact Spectronaut parameters are available via ProteomeXchange, accession no. PXD034772. For identification, a FDR threshold of 0.01 and unspecific digestion rule were used. For targeted DIA-based identification of the peptides, the library was matched against the immunopeptidomics DIA raw files with a *q*-value cut-off of 0.01 and 1, respectively, for precursor and protein. Results from Spectronaut were exported in peptide-centered file formats. These data were used for Figs. 1 and 4–6 and for calculating the sampling scores (Supplementary Tables 2 and 3).

For more extended analysis of HLA-I peptides derived from noncanonical sources, we used the database of translated nuORFs across tissues (nuORFdb)^28^ (concatenated with the human reference proteome; 323,848 entries, PA_nuORFdb_v1.0.fasta) and a reduced version of the above-mentioned personalized references per patient, where the ORF noncanonical sources were restricted to methionine-to-stop, in silico translated, transcript entries, resulting in fasta files with overall a similar size per patient (ranging from 521,779 entries for patient 02672 to 599,300 entries for patient 02287). We used the hybrid DIA approach with Spectronaut v.16.3. Peptide identification was performed by Pulsar on DIA and DDA files separately per patient using unspecific digestion and with a peptide length from 8 amino acids to 15 amino acids. Acetylation at the protein amino terminus and oxidation of methionine were considered as variable modifications. For annotation of nuROF sources, in case a peptide matched multiple nuORF hits, the priority was given with the following order: 5′-uORF, out-of-frame, 3′-dORF, noncoding (nc)RNA and ‘others’. For noncanonical sources, we used the gencode annotation with the following order of priorities: lncRNAs, processed transcripts, pseudogenes, retained introns, noncanonical ORFs and ‘others’ (Supplementary Table 9). In addition, we downloaded the HLA-I and HLA-II files of the HLA atlas^29^ and searched them against the above nuORF fasta file concatenated with all the entries from which we identified noncanonical peptides in our initial analyses, to obtain information about their detection in benign tissues. In the present study, we used the NewAnce tool as mentioned above on an HPC cluster. Identified peptides were aligned against the National Center for Biotechnology Information’s human reference proteome that contains 845,586 entries, including nonidentical sequences from GenBank CDS (protein coding sequence) translations (ncbi.nlm.nih.gov/genbank), Protein Data Bank (PDB; rcsb.org), Uniprot, PIR (proteininformationresource.org) and PRF (prf.or.jp). We regarded leucine and isoleucine as equal. Only entries that did not match any protein in this larger reference were used for further analyses. These data were used for Extended Data Figs. 7–9 and Supplementary Table 9.

### HLA-binding prediction for mass spectrometry-identified peptides

The binding affinity of HLA-I and HLA-II peptides was predicted by the MixMHCpred.v.2.0.2 and MixMHC2pred.v.1 algorithms, respectively, using patient-specific allotypes as determined by HLA typing^49–51^. HLA-I 9-mers and HLA-II 15-mers were supplied as input for this prediction. Peptides with a predicted binding rank ≤2% were considered as binders. Clustering was performed using MixMHCp 2.1 (refs. ^50,52^) on 5,000 randomly selected HLA-I 9-mers from protein-coding sources, and for all noncanonical 9-mers in samples with >100 peptide identifications.

### GTEx RNA expression analyses and listing TAA genes

Tissue-specific gene expression data were downloaded from the GTEx project v.7 (ref. ^53^). The 90th percentile per tissue type in GTEx was reported in TPM values. For the selection of cancer-specific, TAA protein-coding and noncanonical genes, we first listed genes with expression level <1 TPM in any healthy tissues in GTEx (except tesies) and then retained genes with an expression level <1 TPM in any of the healthy macro-regions of our cohort and expression >1 TPM in any of the cancer macro-regions.

#### PCA and cancer types

We used a curated list of known genes that define the three different cancer types (Supplementary Table 4). The PCA was carried out using the ‘svd’ function of base R decomposing the expression matrix of selected genes: *X* *=* **U***D***V**′, with two vectors **U** and **V**′ containing the left and right singular vectors of *X*, and the matrix *D* with non-negative eigenvalues *d*_*i*_; the fraction of explained variance (FOV) is then calculated as: ${\mathrm{FOV}} = \frac{{d^2}}{{\mathop {\sum }\nolimits_{i = 1}^I {{d}}_{{i}}^2}}$.

### Phylogenetic trees and mutational signature deconvolution

For each patient, high-confidence somatic mutations (detected by at least two of the variant callers) were selected and the presence of all mutations and their noncorrected VAFs were assessed in each sample-specific alignment file (BAM) with pysam, minimum_base_quality = 30, minimum_mapq = 20 (https://pysam.readthedocs.io/en/latest/index.html). Tumor content and copy numbers were estimated with Sequenza (v.3.0.0)^54^ and used with noncorrected VAF for the calculation of the cancer cell fraction (CCF) by Palimpsest^55^. CCF/2 was used as the VAF input for LICHeE^56^ and the best scoring tree was selected for each sample.

For each sample, contributions of mutational signatures were deconvoluted using Palimpsest R package (https://github.com/FunGeST/Palimpsest; deconvolution_fit algorithm) on all detected high-confidence somatic mutations. Mutational signature contributions were calculated as the mean contribution of each signature of all samples. Patients with a contribution of SBS4 (associated with tobacco smoking) >50% were categorized as smokers. Hierarchical clustering of the patients was based on the proportions of private, shared and truncal mutations, using R packages dist (method = ‘euclidean’) and hclust (method = ‘ward.D2’).

### HLA sampling density score

HLA sampling density was calculated using the list of identified peptides based on refs. ^57,58^ of each source protein as $D = \frac{K}{{L - 8}}$ for HLA-I and $D = \frac{K}{{L - 14}}$ for HLA-II with *L* the length of the protein, $K = \mathop {\sum}\nolimits_{k = 0}^n {P\left( {x|N\left( x \right)} \right)}$ with *P* the probability to obtain peptide x: $P\left( {x|N} \right) = 1 + \left( {1 - q} \right)^N$ and *N*|*x* the number of protein sequences sharing peptide x; *q* is the a priori expected value of peptides that can be generated by a protein and is set to 0.2.

### Correlations and variance

For correlations of linear models we use the standart ‘cor’, ‘cor.test’ and ‘lm’ function of the R package ‘stats’. For the correlation matrix in Fig. 3h and Extended Data Fig. 3c we used the standard settings of the R package ‘corrplot’. Variance in Fig. 3i and Extended Data Fig. 3d,j was calclulated across all cancer bulk RNA-seq samples per patient with the standard R ‘var’ function and then for the final quantification in Fig. 3j and Extended Data Fig. 3e averaged across all micro-regions per patient.

### Calculation of inflammation scores and cell-type abundance

We computed an inflammation score based on the procedure outlined in Danaher et al.^17^. This signature is used in Fig. 2c,d for each macro-region based on bulk RNA expression. For the quantification of immune cell types, we used the signature of Danaher et al.^17^, and defined the cell-type abundance as the mean of the log_2_(expression values) of all annotated and selected genes per cell type, which were also measured in the GeoMx transcriptome atlas.

### HLA presentation hotspots and prediction of neoantigens

The ipMSDB database^34^ from assembly of 1,102 immunopeptidomic raw files searched with Comet (PSM FDR of 1%) was used as previously described^47^. None of raw files of the investigated eight patients with lung cancer from the present study were included in this version of ipMSDB.

For neoantigen prediction, only ‘high-confidence’ calls were selected, defined as the set of variants containing all somatic nonsynonymous, synonymous mutations and phased SNPs detected by at least two of the variant callers described above. MixMHCpred.v.2 (ref. ^49^) was run on all predicted 9-mer to 12-mer neoantigen peptides covering nonsynonymous and synonymous somatic mutations in each macro-region using patient-specific HLA allotypes. Neoantigens with a predicted binding rank ≤2% were considered as binders. The overlap of the wild-type counterparts of the predicted neoantigen with all other HLA-I peptides in ipMSDB was determined. Neoantigens identical to wild-type sequences in SwissProt^59^ or found in the reference GRCh37 (ref. ^43^) proteome were filtered out. We calculated the fraction of ‘exact’ matches as $F_{{\mathrm{ex}}} = \frac{{{{N}}_{{{{\mathrm{ex}}}}}}}{{{{N}}}}_{{{{\mathrm{total}}}}}$ with *N*_ex_ the number of ‘exact’ match peptides and *N*_total_ the total number of neoantigens passing the filter for binding. To correct for potential biases due to the availability of some HLA alleles in ipMSDB, we used the same approach to analyze synonymous mutations. These are assumed to not be subjected to immune pressure. For those, we calculated the same fraction as before, $F_{{\mathrm{ex}},{\mathrm{syn}}} = \frac{{{{N}}_{{{{\mathrm{ex}}}},{{{\mathrm{syn}}}}}}}{{{{N}}_{{{{\mathrm{total}}}},{{{\mathrm{syn}}}}}}}$ this time with *N*_syn_ the total number of predicted binders covering synonymous mutations and *N*_ex,syn_ the fraction of peptides that are binders and also map to ‘exact’ matches. The enrichment was then defined as *F*_ex_/*F*_ex,syn_.

For the enrichment in Fig. 8h–n we calculated the fraction of ‘exact’ matches of neoantigens predicted for nonsynonymous and synonymous mutations per sample. Missing values were imputed as the minimal value of each annotated group. We excluded macro-regions 02289-08 and 02289-09 because no synonymous mutations were found.

### Analysis of published datasets

The TRACERx data files were downloaded from the European Genome phenome Archive (EGA) archive (accession numbers EGAD00001004591 and EGAD00001003206). We included all patients for whom both WES and RNA-seq data were available. The mapped bam files were converted to fastq with samtools and mapped to GRCh37 with bwa. We reduced the file size of the resulting fastq files to 50% of the original size. HLA typing was predicted using arcasHLA^60^. The data were analyzed in the same way as the lung cohort described above. We excluded samples CRUK0079-R3 due to RNA-seq pipeline errors, CRUK0004 because no synonymous mutations were found and CRUK0012 because only three alleles were available for predictions and no synonymous mutations predicted to be binders to the patient’s HLA were found. Light smokers were assigned as those with a contribution of tobacco smoking signature of a maximum 30%, whereas heavy smokers were those with at least a 70% smoking signature. Heavy smokers additionally were required to have ≥70 pack-years.

We similarly downloaded and analyzed the dataset from the National Cancer Institute (NCI) Surgery Brunch published by Gartner et al.^35^ where mutations were screened for immunogenicity with the mini-gene approach. Out of 81 patients, 77 with at least one mutation were found to be immunogenic. We filtered out four patients—2098, 3309, 1913 and 2224—according to Gartner et al.^35^. In total, 132 mutations were annotated as ‘immunogenic’. For all high-confidence-called somatic mutations, neoantigens were predicted and filtered for binders as described above. Predicted neoantigens were annotated as ipMSDB ‘exact’ and ‘nonexact’. We further calculated the fraction of ‘exact’ and ‘nonexact’ matches in ‘immunogenic’ and ‘nonimmunogenic’ mutations to learn the probability of mutations being immunogenic, depending on their classification into ‘exact’ (*f*_ex_) or ‘nonexact’ (*f*_nonex_).

To estimate the immunogenic potential of a mutation in our cohort, we calculated the relative immunogenicity, which is the probability of a mutation being immunogenic when sampled randomly for a given patient: ${\mathrm{Relative}}\,{\mathrm{immunogenicity}} = \frac{{{{N}}_{{{{\mathrm{ex}}}}} \times {{f}}_{{{{\mathrm{ex}}}}} + {{N}}_{{{{\mathrm{other}}}}} \times {{f}}_{{{{\mathrm{nonex}}}}}}}{{{{N}}_{{{{\mathrm{total}}}}}}}$ with *N*_total_ the total number of mutations, *N*_ex_ and *N*_nonex_ the number of ‘exact’ and ‘nonexact’ matches per macro-region and *f*_ex_ and *f*_nonex_ the learned values above. We quantified the significance of the difference in relative immunogenicity using a standard Wilcoxon’s test between smokers and nonsmokers.

### Correlation of immune cell abundance and GeoMx gene expression

For all source genes detected in the HLA-II peptidome in each tumor group that were also measured in the GeoMx CTA, we calculated a *z-*score for each gene *i* between tumor and stroma, CD45^+^ and TLS regions (called stroma) related: $z_i = \frac{{\left\langle {R_{{\mathrm{t}},i}} \right\rangle - \left\langle {R_{{\mathrm{s}},i}} \right\rangle }}{{\sqrt {{\mathrm{var}}\left( {R_{{\mathrm{t}},i}} \right) + {\mathrm{var}}\left( {R_{{\mathrm{t}},i}} \right)} }}$, with *R*_t*,i*_ and *R*_s*,i*_ the log_2_(expression values) of gene *i* in tumor (t) or stroma (s) regions. We then subselected those genes with a *z*-score that falls into the 25th (stroma) and 75th (tumor) percentiles. We correlated the expression of those genes with the previously estimated immune cell-type abundance, across all micro-regions in the respective tumor group, to find genes with expression associated with the immune compartment. For correlations, we used the standard ‘cor’ function of base *R*, with the default method ‘Pearson’. To further associate immune cells with the presentation of those genes, we summed up the mean sampling scores of all genes with a correlation >0.5 per cell type. $S_c = \mathop {\sum}\nolimits_{j = 1}^J {\frac{1}{G}} \mathop {\sum}\nolimits_{g = 1}^G {{{P}}_{{{{\mathrm{gj}}}}}}$ with *P* the presentation score of gene *j* in group *G*.

### One- and two-dimensional GO analysis

GO enrichment analysis was carried out on all genes using the R package ‘TopGO’. Our gene universe contained all genes expressed and measured in the GeoMx CTA. We selected genes to be highly expressed in HLA-II^+^ or HLA-II^−^ samples by calculating a *z*-score $z = \frac{{\left\langle {{\mathrm{HLAII}} ^+ } \right\rangle - \left\langle {{\mathrm{HLAII}} ^- } \right\rangle }}{{\sqrt {{\mathrm{var}}\left( {{\mathrm{HLAII}} ^- } \right) + {\mathrm{var}}\left( {\mathrm{{HLAII}} ^+ } \right)} }}$ and a fold-change (FC) ${\mathrm{FC}} = \frac{{\left\langle {{\mathrm{HLAII}} ^+ } \right\rangle }}{{\left\langle {{\mathrm{HLAII}} ^- } \right\rangle }}$ on the log_2_ transformed GeoMx CTA gene expression data. Our selection of genes for the enrichment analysis contained the genes that differ significantly between the groups based on the *z*-score: *z* > 2(HLA-II^+^) or *z* < −2(HLA-II^−^).

For the 2D GO analysis^61^ log_2_(fold-changes) (log_2_(FC)) between two groups of tumor samples (high, low and infiltrated, excluded) were calculated along with differential gene expression analysis using the R package ‘edgeR’ on the bulk RNA-seq expression data (raw counts). Source genes were ranked according to their log_2_(FC) (high–low) and (infiltrated–excluded). We then annotated all source genes with GO categories without thresholds using the R package TopGO (2.40.0). The scores *s*_*x*_ and *s*_*y*_ for both comparisons were then calculated for all GO categories: $s_{x,y} = \frac{{2\left( {R_g - R_o} \right)}}{n}$, with *R*_*g*_ the mean rank in the respective GO category and *R*_*o*_ the rank in all the other GO categories. To simplify the display, we selected terms that fall into $\mathop { \to }\limits_s = \sqrt {s_x^2 + s_y^2} > 0.3$. For 2D GO analysis on the HLA sampling scores in Extended Data Fig. 4b, we used the same approach but the differences between both groups were assessed by calculating a *z*-score for two comparisons $z_C = \frac{{\left\langle {S_{c1}} \right\rangle - \left\langle {S_{c2}} \right\rangle }}{{\sqrt {{\mathrm{var}}\left( {S_{c2}} \right) + {\mathrm{var}}\left( {S_{c1}} \right)} }}$, with *S*_*c*1_and *S*_*c*2_ the groups of both comparisons, that is, *S*_*c*_ = high, *S*_*c*2_ = low and infiltrated/excluded, on the sampling scores. We then ranked the genes by the *z*-score and applied the GO analysis in the same manner as for the RNA expression analysis. For simplicity we here displayed only terms with a distance to origin >0.2: $\mathop { \to }\limits_s = \sqrt {s_x^2 + s_y^2} > 0.2$.

### Selection of marker genes in the HLA-II peptidome

We selected all GO categories from the 2D space above with a distance from origin >0.2 and filtered them according to their sampling score. We retained genes for both of our comparisons: genes presented in ≥50% of replicates in immune-low or immune-high samples (inflammation), and genes presented in ≥50% of the replicates in excluded or infiltrated samples (infiltration).

### Presentation efficiency

We selected all macro-regions that were measured both by RNA-seq and HLA-I DIA peptidomics and filtered for expressed TAAs within each macro-region. Then, we calculated, for each TAA in each macro-region, the presentation efficiency *P*_eff_ as: $P_{{\mathrm{eff}}}\left( i \right) = \frac{{P_i}}{{E_i \times \left( {1 - \frac{1}{{E_{{\mathrm{HLA}}} + \varepsilon }}} \right)}}$, with *P*_*i*_ the sampling density score, *E*_*i*_ the expression of TAA, *i* and *ε* the detection limit in the GeoMx CTA atlas, set to the 0.1th percentile of the detected values for all measured genes. TAAs with sampling score equal zero were included to factor in expressed yet nonpresented TAAs. We normalize this fraction by *E*_HLA_, the mean expression of the three HLA genes HLA-A, HLA-B and HLA-C in the GeoMx CTA (tumor micro-regions). To obtain the mean presentation efficiency for each macro-region, we then calculated $\left\langle {P_{{\mathrm{eff}}}} \right\rangle = \frac{1}{N}\mathop {\sum}\nolimits_{i = 1}^I {P_{{\mathrm{eff}}}\left( i \right)}$, where *N* is the total number of expressed TAAs in a macro-region.

### Neoantigen quality model

We calculated the quality *Q*_*i*_ for all predicted binders per region *i* as outlined in Łuksza et al.^32^. Neoantigens were grouped according to their respective mutations being truncal (found in at least (no. of regions − 1) regions), private (maximum 2 regions) and clonal (if not assigned to any of these two categories). Due to low mutational load the following samples were treated differently. A mutation was assigned as truncal when found in no. of regions − 2 in 02672 and 02287, or no. of mutations − 3 in 02671 and 02289. We calculated the quality changes *q* due to immune editing: $q_i = \frac{{\left\langle {{{{\mathrm{Q}}}}_{{{{\mathrm{truncal}}}},{{i}}}} \right\rangle }}{{\left\langle {{{{\mathrm{Q}}}}_{{{{\mathrm{private}}}},{{i}}}} \right\rangle }}$ with *i* iterating over each region.

### Statistics and reproducibility

No statistical method was used to predetermine sample size. No patients or macro-regions of the eight lung cohort patients were excluded throughout the analysis. From the TRACERx cohort, we excluded from the analysis samples CRUK0079-R3 due to RNA-seq pipeline errors, CRUK0004 because no synonymous mutations were found and CRUK0012 because only three alleles were available for predictions and no synonymous mutations predicted to be binders to the patient’s HLA were found. In the NCI dataset, we excluded patients 1913, 2098, 2224 and 3309 because, for those, only immunogenic mutations were included in the dataset^35^. Data collection and analysis were not performed blind to the conditions of the experiments. Data were not randomized. Data distribution was assumed to be normal but this was not formally tested. The mIF, GeoMx, WES, RNA-seq and immunopeptidomic experiments were performed only once.

Statistical analyses were performed where applicable using standard applications in R 4.0.2. For all boxplots, we used the standard setting of the package ‘ggplot2’. Boxplots do not display confidence intervals (CIs), the degrees of freedom are standard for two sample tests, *n* − 2 with *n* the sample size. Effect sizes were not considered. Correlation and corresponding *P* values in Fig. 5e,g were assessed with standard cor and cor.test functions of the R ‘stats’ package. The correlation matrices in Figs. 3h and 5h,i were calculated and plotted using the R package ‘corrplot’. All corresponding tests that supply a *P* value were mentioned in the figure legends. Further information on research design is available in the Nature Research Reporting Summary linked to this article.

### Ethical regulation

Informed consent was obtained from the participants in accordance with the requirements of the institutional review board (Ethics Commission, Centre hospitalier universitaire vaudois (CHUV), Lausanne, Switzerland and Bioethics Committee, Poznań University of Medical Sciences, Poznań, Poland).

### Reporting summary

Further information on research design is available in the Nature Portfolio Reporting Summary linked to this article.

## Supplementary information

Supplementary Information Supplementary Fig. 1.

Reporting Summary

Supplementary Table Supplementary Tables 1–12.

## Data Availability

The datasets generated and analyzed during the present study are available in the EGA and can be accessed with accession no. EGAS00001006298. mass spectrometry data and Spectronaut parameters are available via ProteomeXchange with accession no. PXD034772. The TRACERx NSCLC WES and RNA-seq data files were downloaded from the EGA archive (accessions EGAD00001004591 and EGAD00001003206). The WES and RNA-seq data of Gartner et al.^35^ were downloaded from dbGap accession no. phs001003v1.p1. All other data supporting the findings of the present study are available from the corresponding author on reasonable request. Source data are provided with this paper.

## Source data

Source Data Fig. 1 A single file containing all source data for all figures and Extended Data figures.

## Code availability

An executable jar file of NewAnce has been deposited to PRIDE with dataset accession no. PXD013649. The NewAnce code is available on the following GitHub link: https://github.com/bassanilab/NewAnce.git.
