# Supplementary material for: The immunopeptidome landscape associated with T cell infiltration, inflammation and immune editing in lung cancer
Source: Nat Cancer. 2023 May 1;4(5):608–28. doi: 10.1038/s43018-023-00548-5 (PMC10212769; doi:10.1038/s43018-023-00548-5)

nature cancer

Article https://doi.org/10.1038/s43018-023-00548-5

The immunopeptidome landscape associated with T cell infiltration, inflammation and immune editing in lung cancer

In the format provided by the authors and unedited

Supplementary Figure 1:

GeoMx ROIs per patient. Selection of defined micro-regions (i.e. regions of interest, ROI) that were selected manually without independent repetition and subjected to spatial analyses (91-plex GeoMx Protein Assay and the Cancer Transcriptome Atlas Design RNA-GeoMx). According to the morphological differences and the above markers, the selected micro-regions were annotated when applicable as either 1. tumoral 2. necrotic, 3. Stroma (with variable contributions of tumor cells and immune cells), 4. $CD45^+$ immune cell rich regions, 5. tertiary lymphoid structures (TLS), and 6. other regions (including blood vessels and non-malignant lung). High resolution images are available upon request.

02287

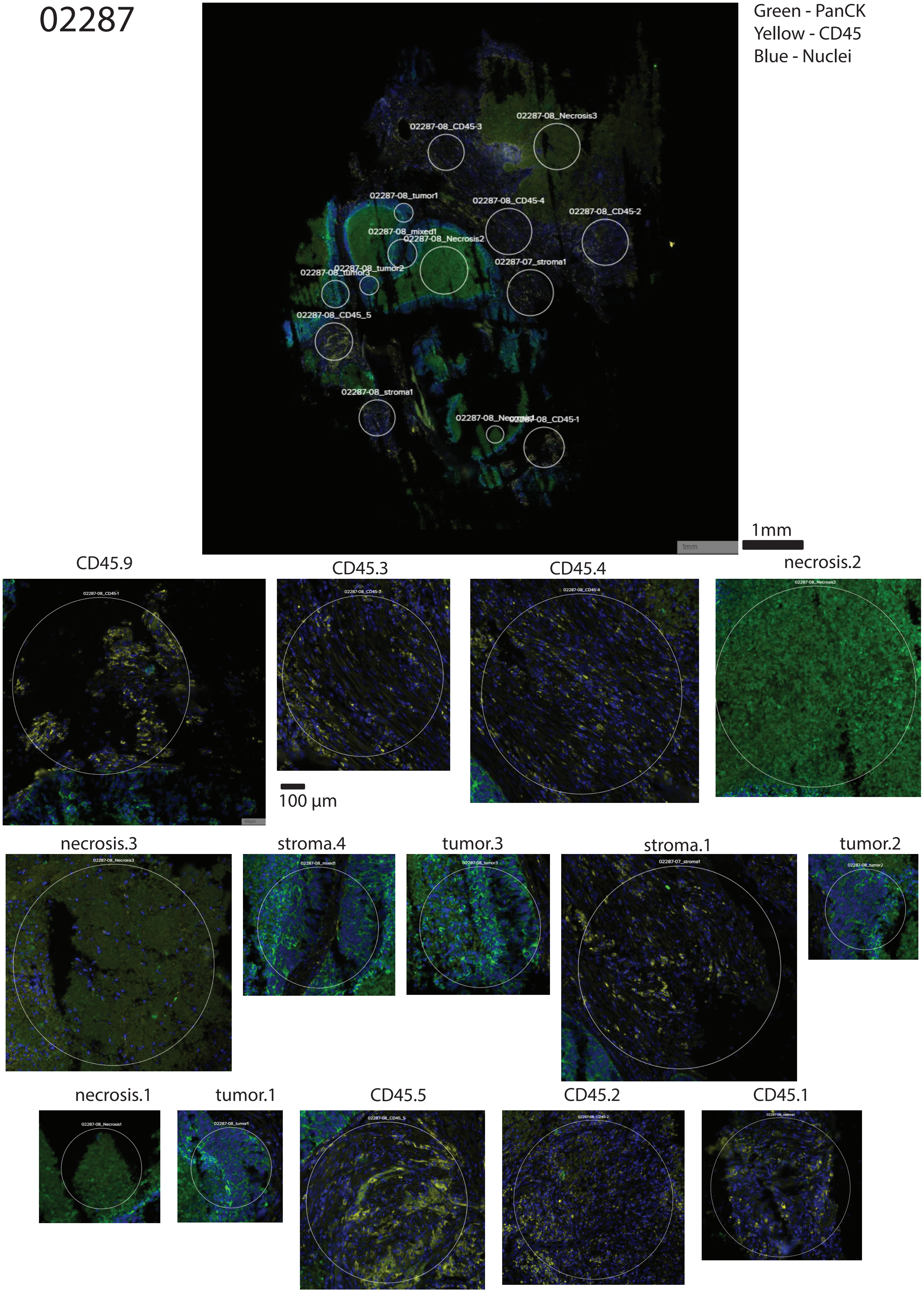
Green - PanCK
Yellow - CD45
Blue - Nuclei
02287-08_CD45-3
02287-08_Necrosis3
02287-08_tumor1
02287-08_CD45-4
02287-08_CD45-2
02287-08_mixed1
02287-08_Necrosis2
02287-07_stroma1
02287-08_tumor2
02287-08_CD45_5
02287-08_stroma1
02287-08_CD45-1
1mm
CD45.9
CD45.3
CD45.4
necrosis.2
100 µm
necrosis.3
stroma.4
tumor.3
stroma.1
tumor.2
necrosis.1
tumor.1
CD45.5
CD45.2
CD45.1

02288

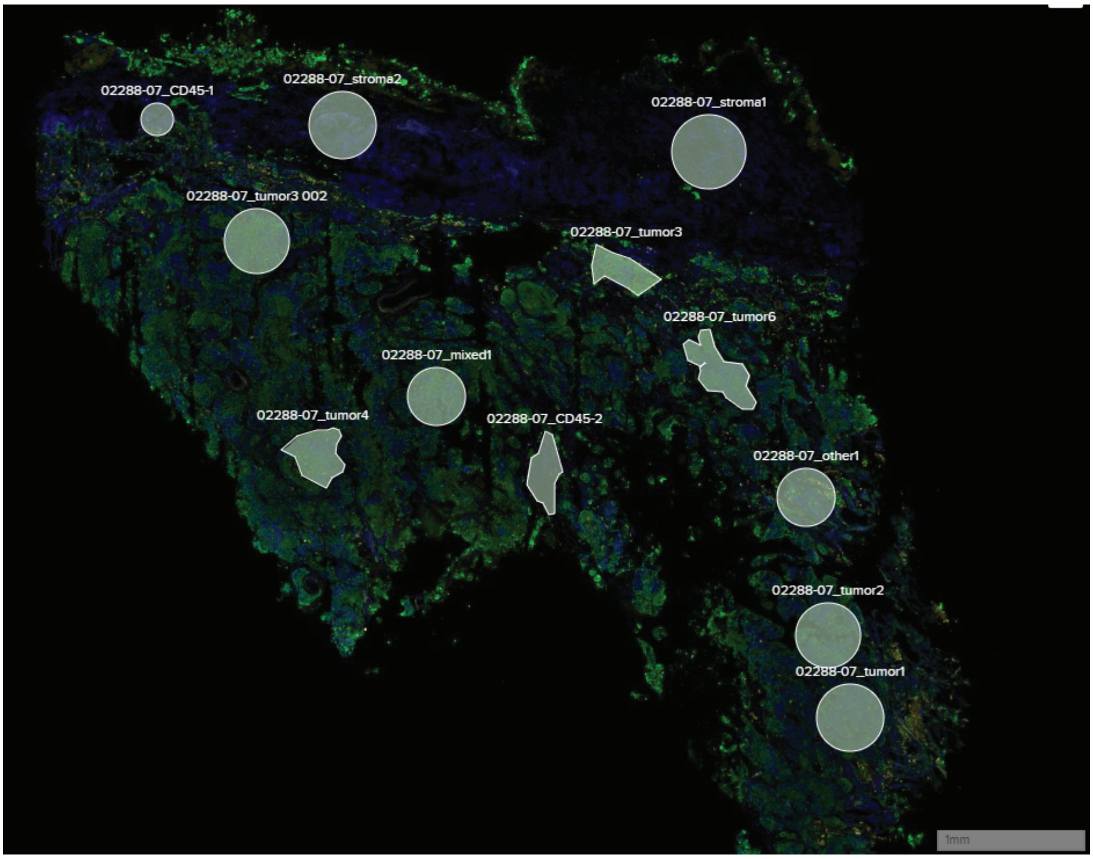

Green - PanCK
Yellow - CD45
Blue - Nuclei

1mm

tumor.3 002

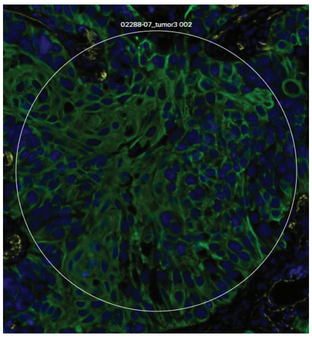

stroma.2

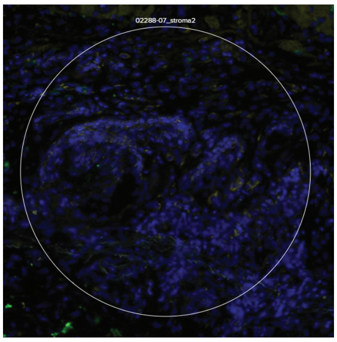

tumor.6

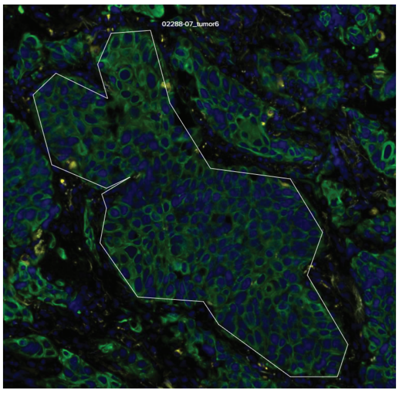

CD45.1

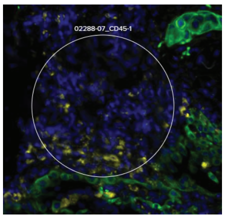

tumor.2

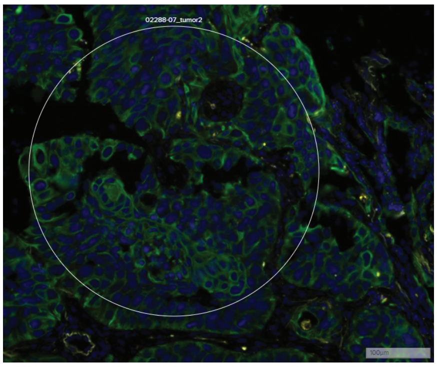

CD45.1

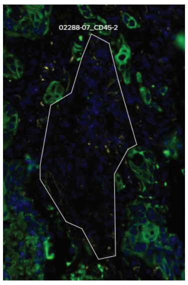

100 µm

tumor.8

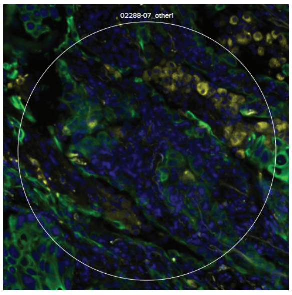

stroma.1

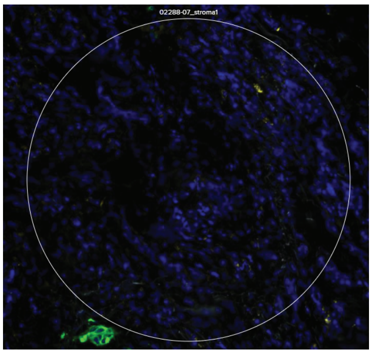

tumor.3

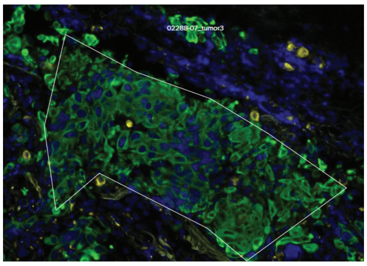

tumor.9

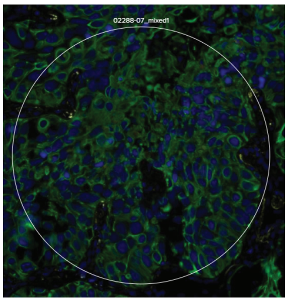

tumor.1

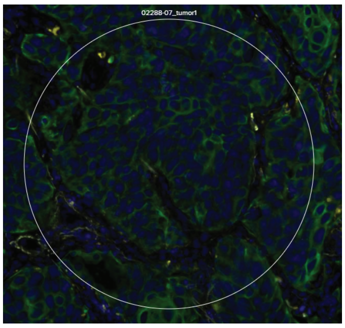

tumor.4

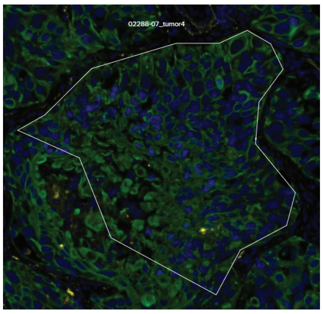

02289

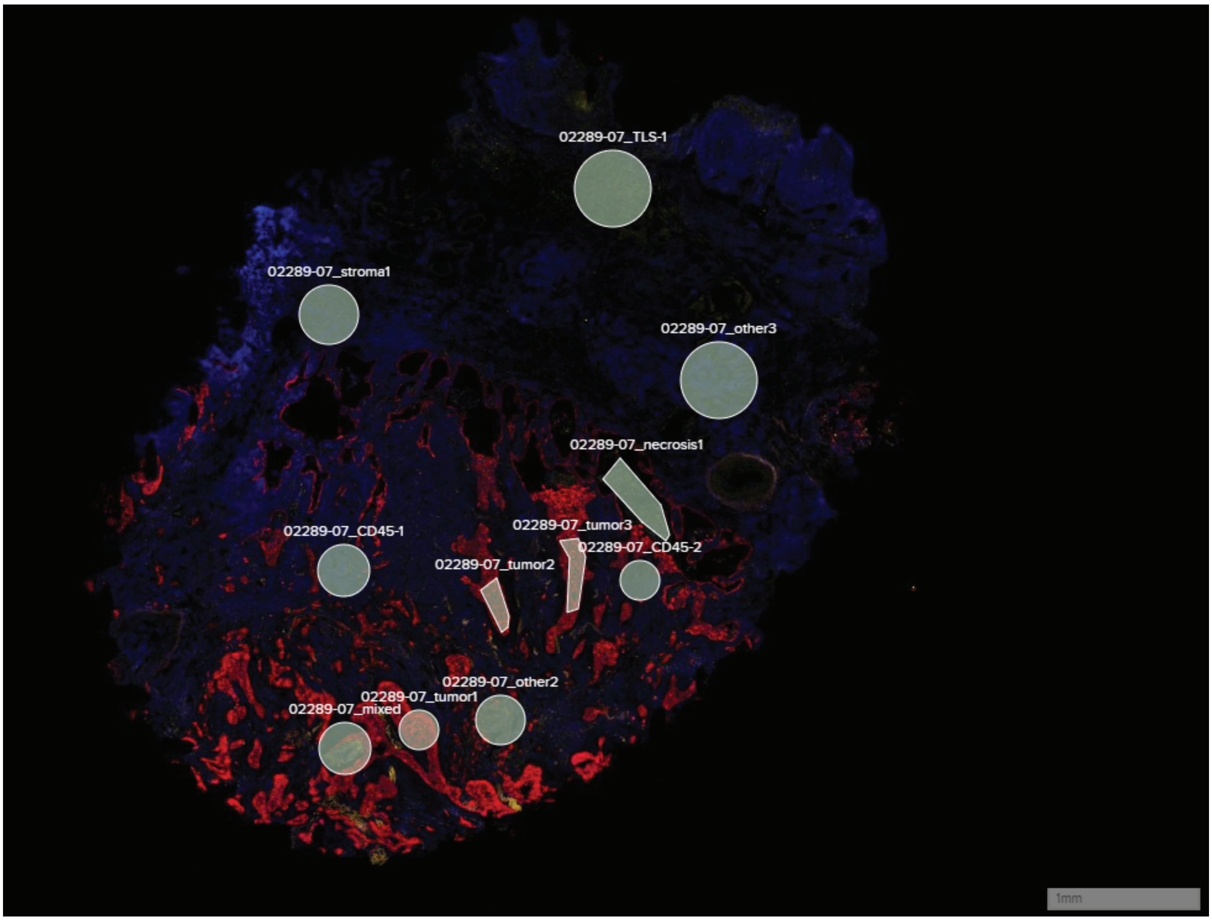

Green - PanCK
Yellow - CD45
Blue - Nuclei

1mm

stoma.1

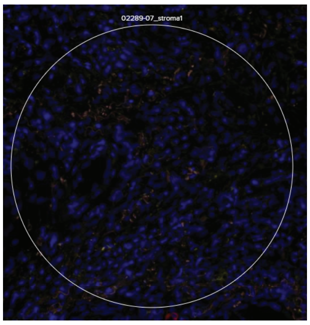

tumor.2

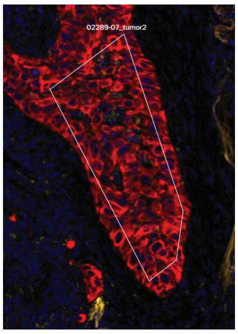

tumor.3

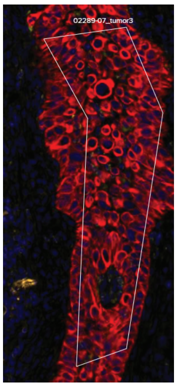

necrosis.1

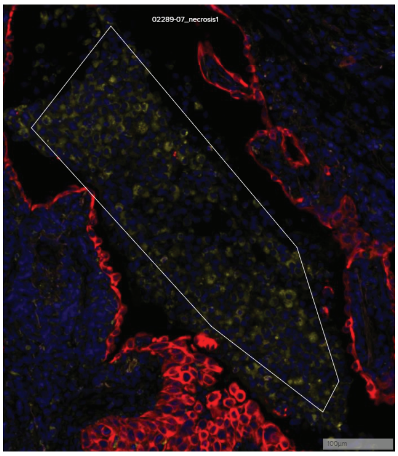

100 µm

other.3

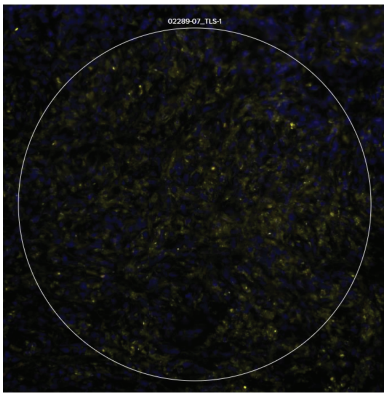

tumor.1

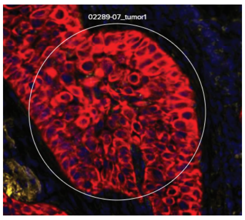

stroma.2

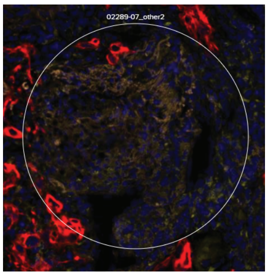

other.3

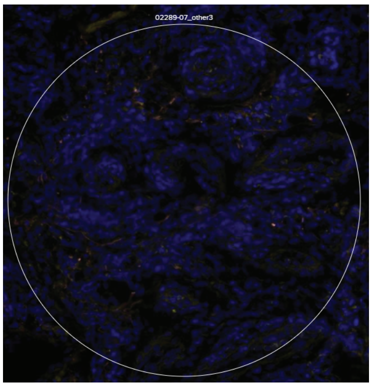

stroma.9

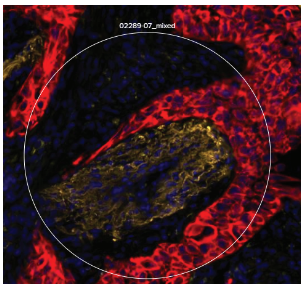

CD45.2

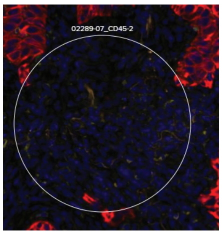

CD45.1

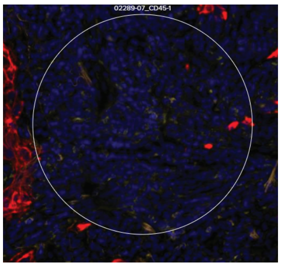

02290

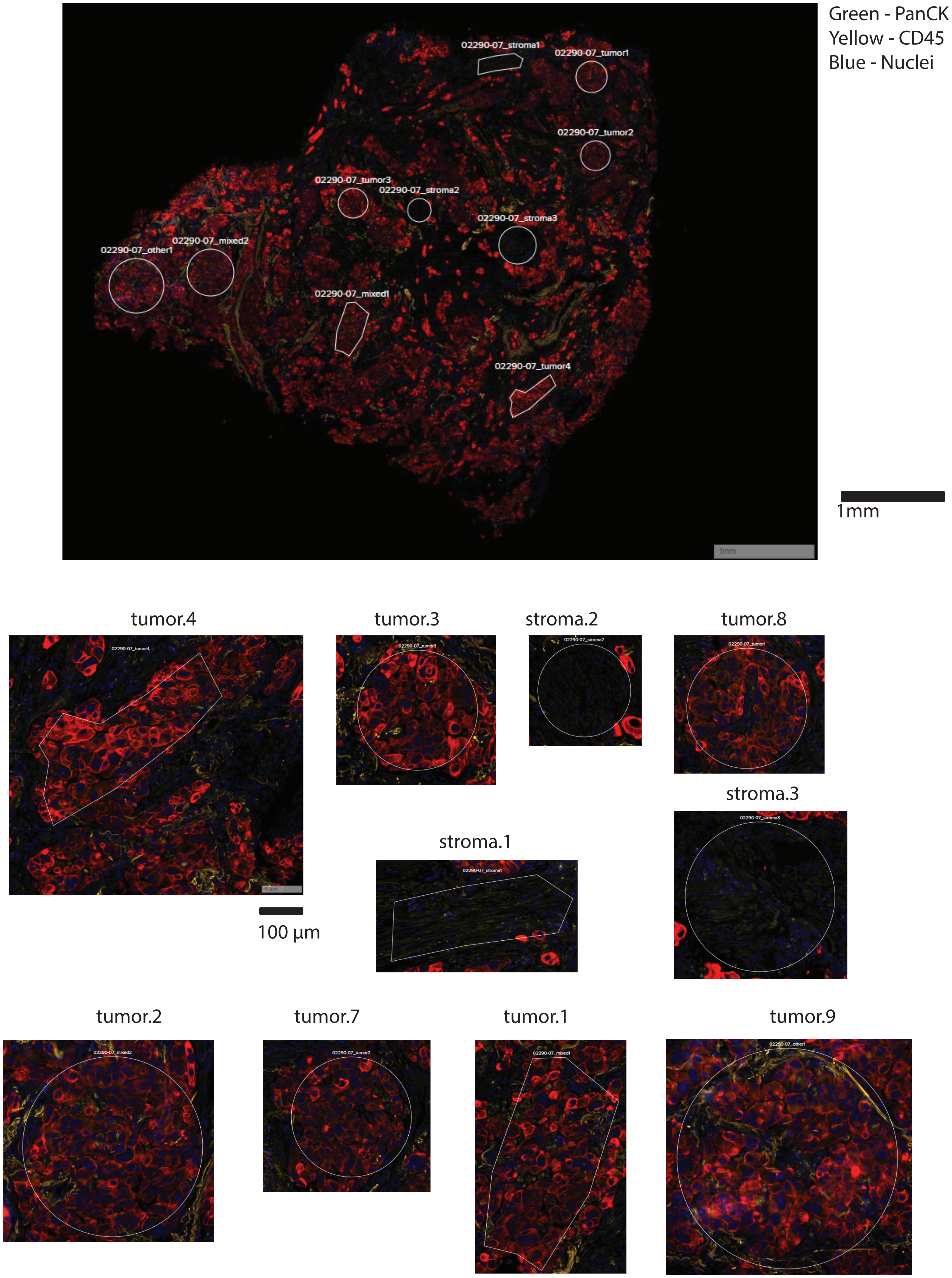
Green - PanCK
Yellow - CD45
Blue - Nuclei
02290-07_stroma1
02290-07_tumor1
02290-07_tumor2
02290-07_tumor3
02290-07_stroma2
02290-07_stroma3
02290-07_other1
02290-07_mixed2
02290-07_mixed1
02290-07_tumor4
1mm
tumor.4
tumor.3
stroma.2
tumor.8
stroma.3
stroma.1
100 µm
tumor.2
tumor.7
tumor.1
tumor.9

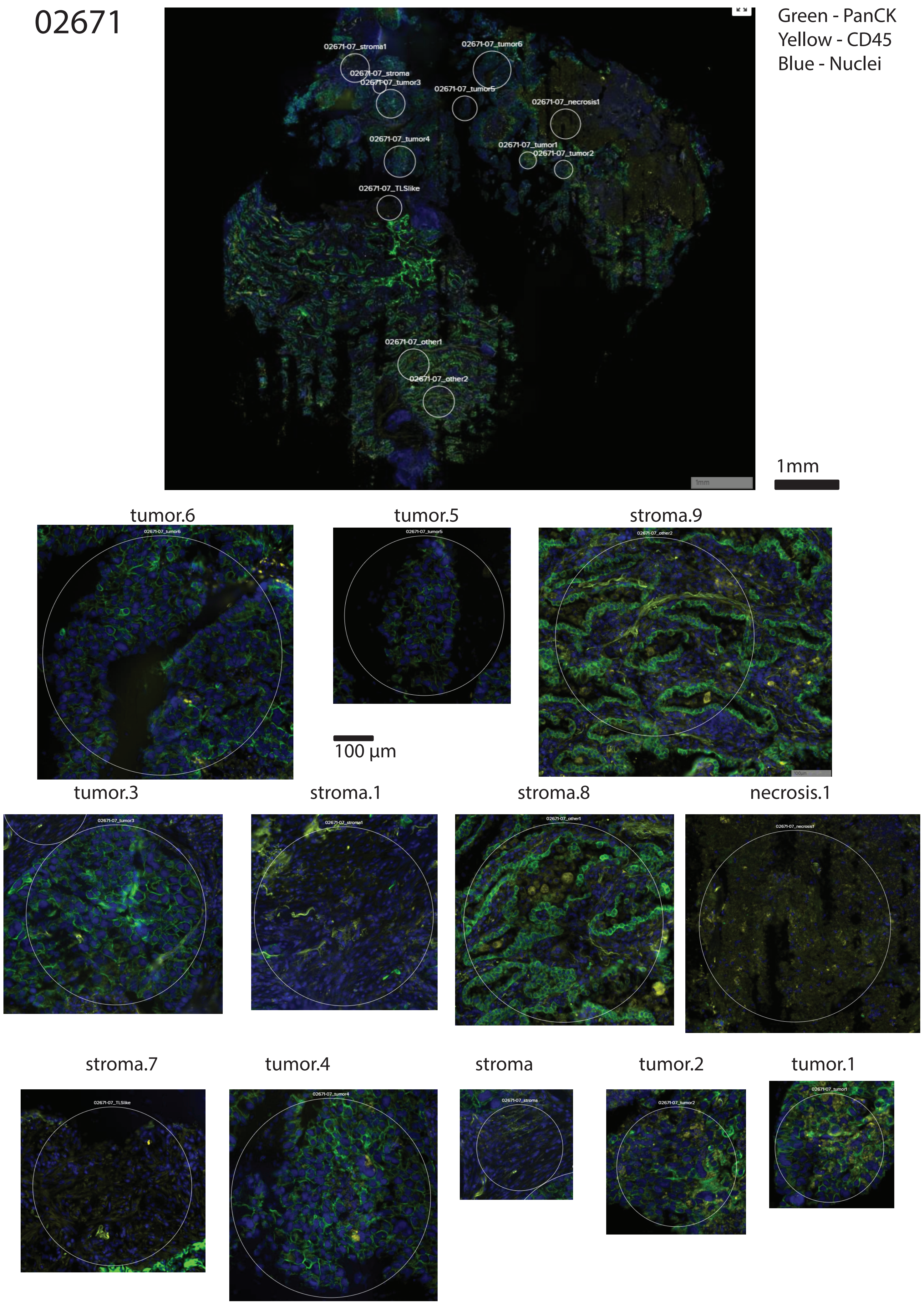

02671
Green - PanCK
Yellow - CD45
Blue - Nuclei
02671-07_stroma1
02671-07_stroma
02671-07_tumor3
02671-07_tumor6
02671-07_tumor5
02671-07_necrosis1
02671-07_tumor4
02671-07_tumor1
02671-07_tumor2
02671-07_TLSlike
02671-07_other1
02671-07_other2
1mm
tumor.6
tumor.5
stroma.9
100 µm
tumor.3
stroma.1
stroma.8
necrosis.1
stroma.7
tumor.4
stroma
tumor.2
tumor.1

02672

Green - PanCK
Yellow - CD45
Blue - Nuclei

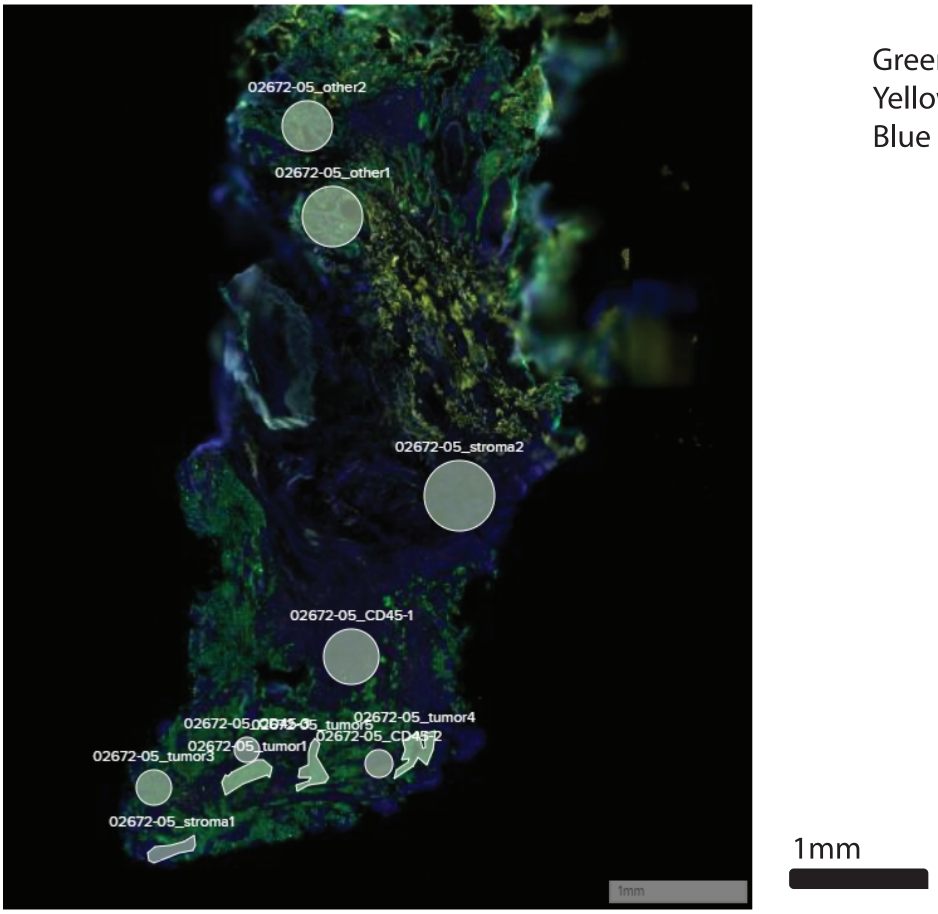

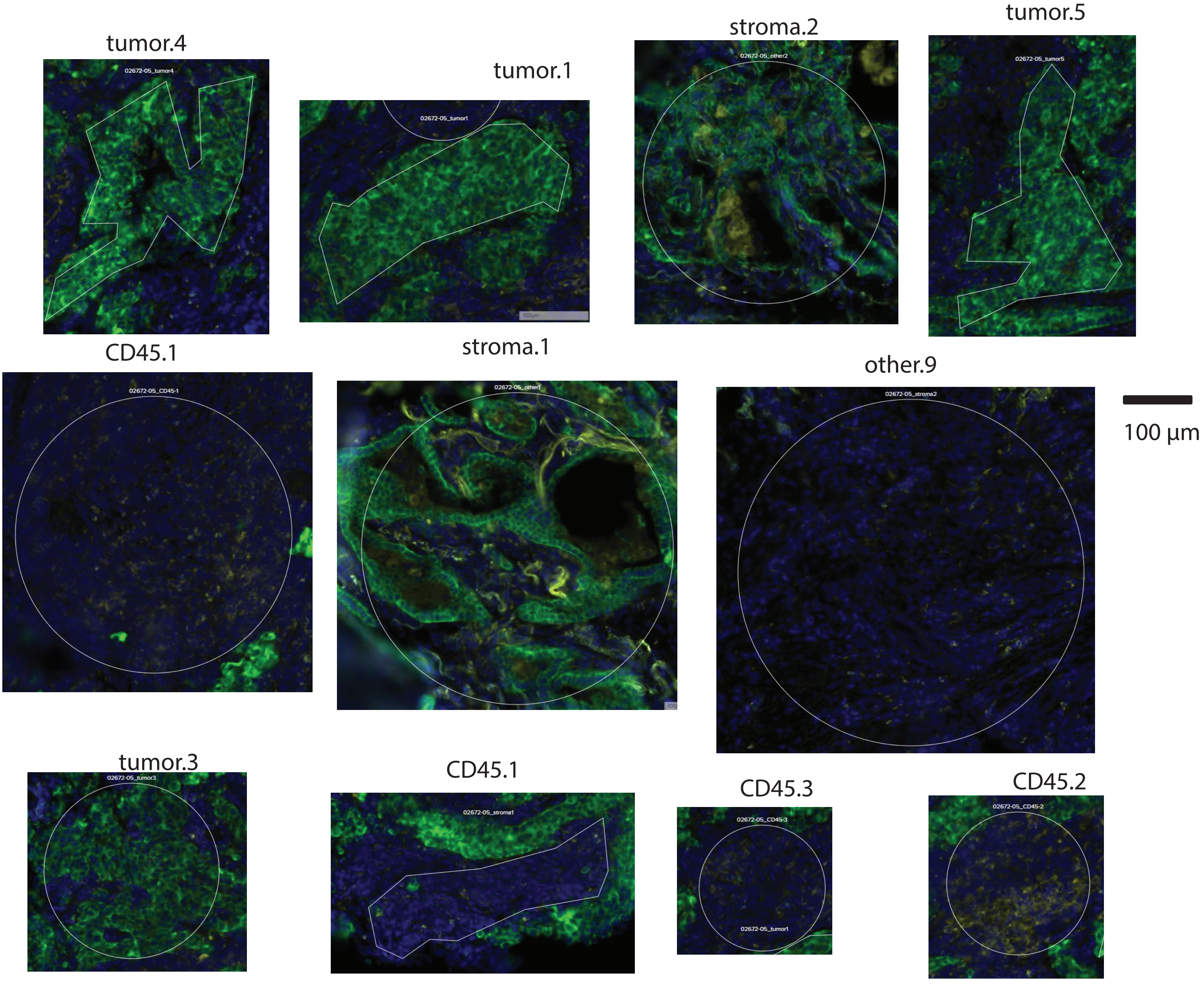

03023

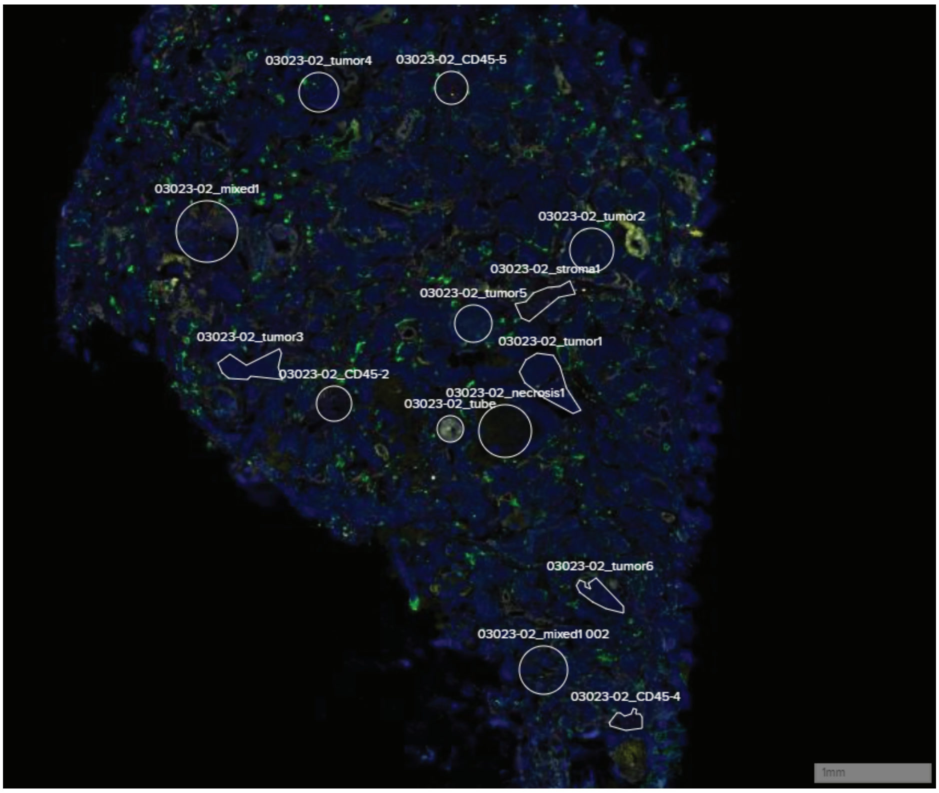

Green - PanCK
Yellow - CD45
Blue - Nuclei

1mm

tumor.7

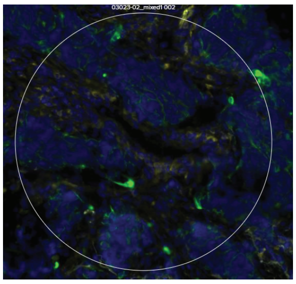

necrosis.1

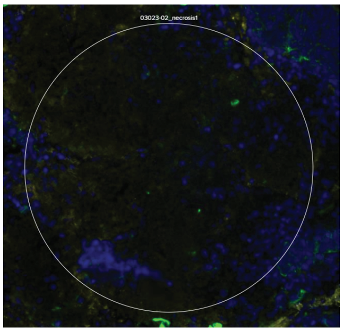

tumor.1

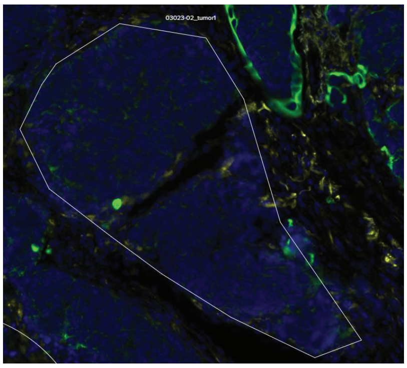

tumor.6

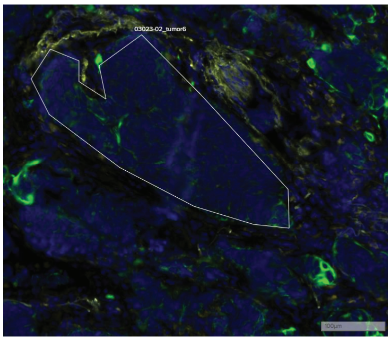

CD45.4

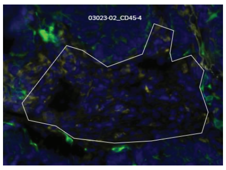

tube

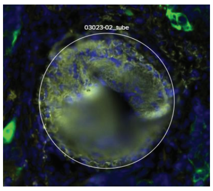

stroma.8

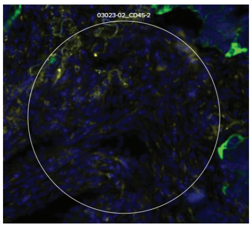

stroma.4

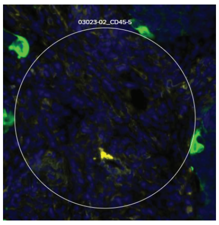

tumor.5

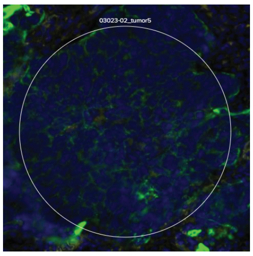

stroma.9

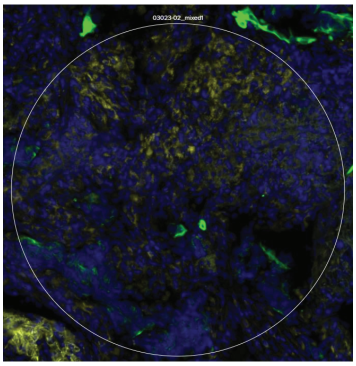

tumor.3

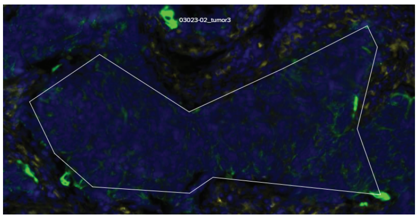

tumor.4

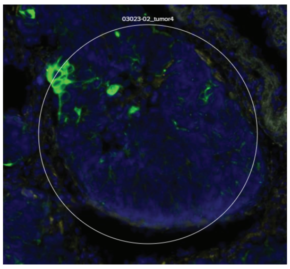

stroma.1

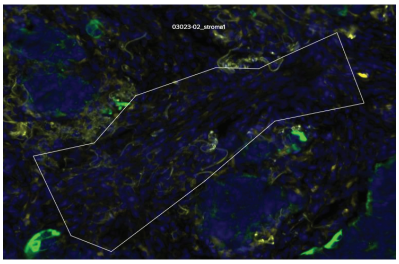

100 µm

tumor.5

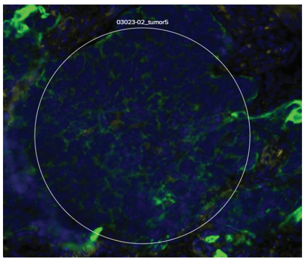

tumor.2

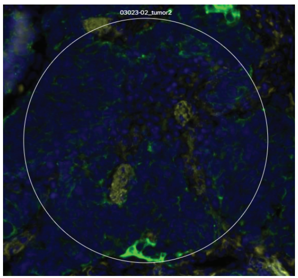

03421

Green - PanCK
Yellow - CD45
Blue - Nuclei

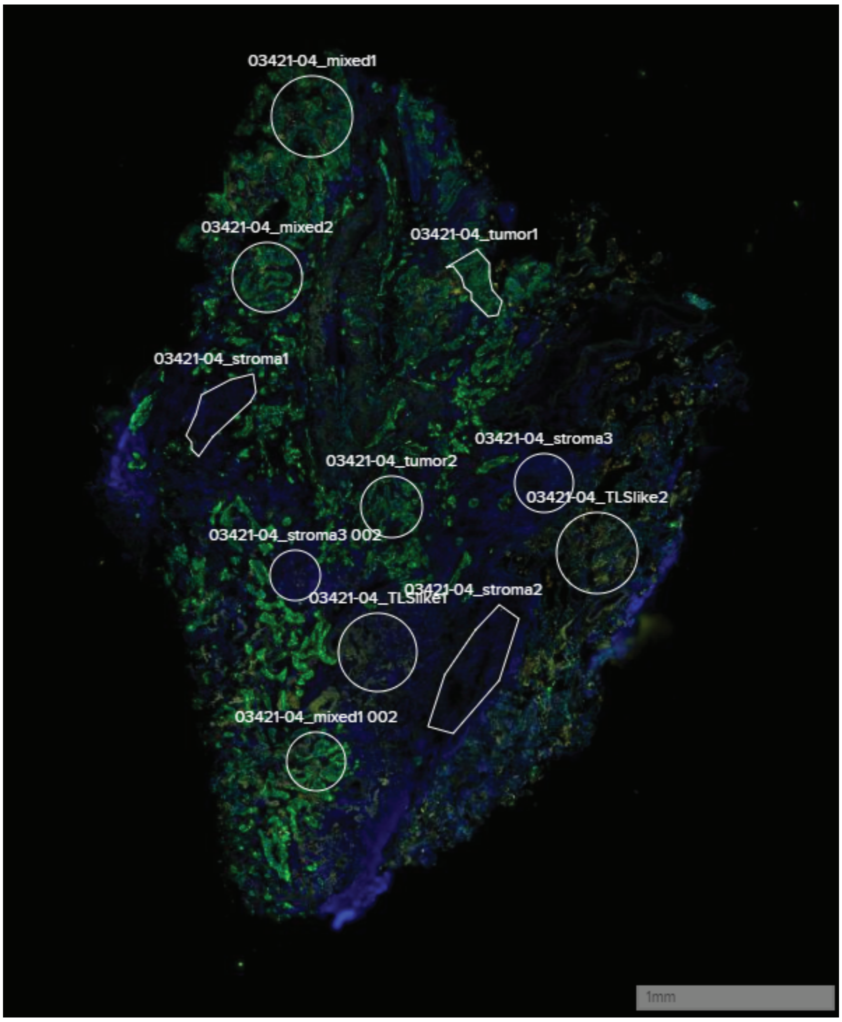

1mm

stroma.1

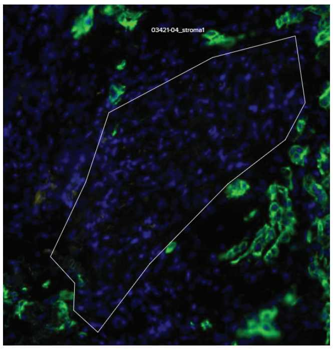

other.2

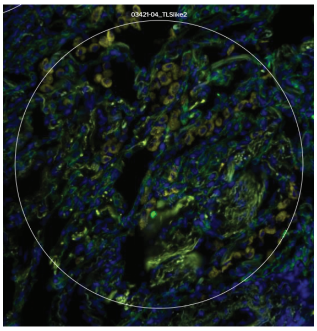

stroma.3

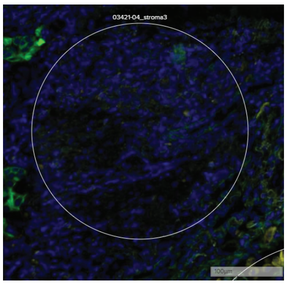

tumor.9

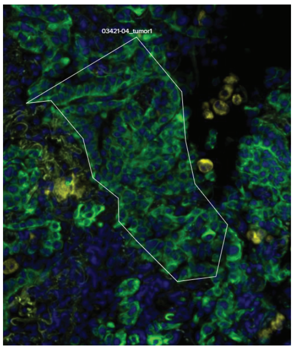

CD45.1

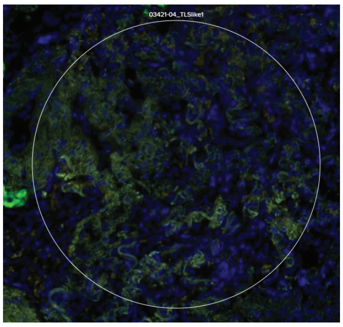

tumor.5

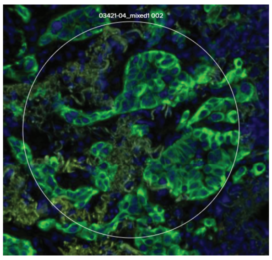

stroma.9

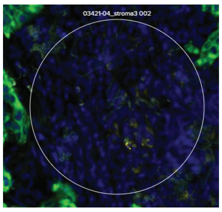

100 µm

stroma.2

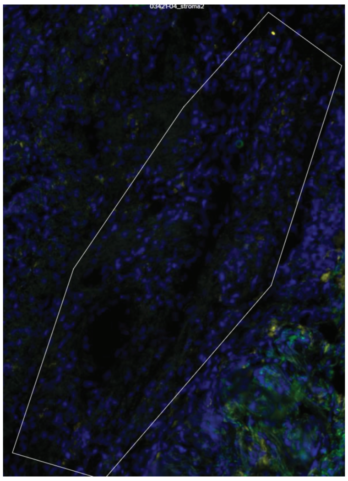

tumor.1

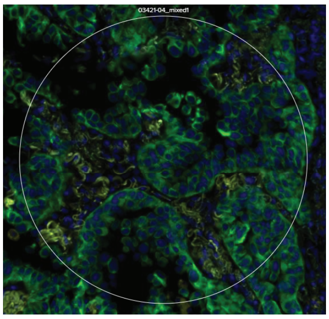

tumor.8

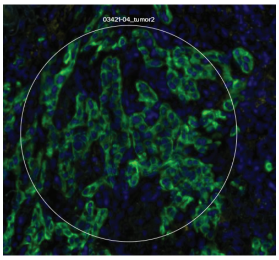

tumor.2

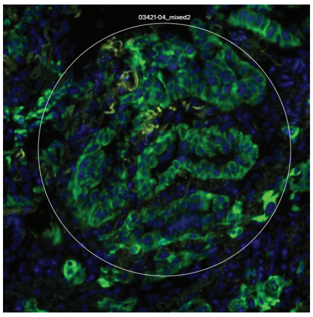

Supplement: Supplementary file 1 — Supplementary Fig. 1. [file 43018_2023_548_MOESM1_ESM.pdf]
