# Supplementary material for: The immunopeptidome landscape associated with T cell infiltration, inflammation and immune editing in lung cancer
Source: Nat Cancer. 2023 May 1;4(5):608–28. doi: 10.1038/s43018-023-00548-5 (PMC10212769; doi:10.1038/s43018-023-00548-5)
Supplement: Supplementary file 2 — Reporting Summary [file 43018_2023_548_MOESM2_ESM.pdf]

nature portfolio

Corresponding author(s): Michal Bassani-Sternberg
Last updated by author(s): 06.03.2023

Reporting Summary

Nature Portfolio wishes to improve the reproducibility of the work that we publish. This form provides structure for consistency and transparency in reporting. For further information on Nature Portfolio policies, see our Editorial Policies and the Editorial Policy Checklist.

Statistics

For all statistical analyses, confirm that the following items are present in the figure legend, table legend, main text, or Methods section.

n/a	Confirmed	
☐	☒	The exact sample size (*n*) for each experimental group/condition, given as a discrete number and unit of measurement
☐	☒	A statement on whether measurements were taken from distinct samples or whether the same sample was measured repeatedly
☐	☒	The statistical test(s) used AND whether they are one- or two-sided<br>*Only common tests should be described solely by name; describe more complex techniques in the Methods section.*
☒	☐	A description of all covariates tested
☐	☒	A description of any assumptions or corrections, such as tests of normality and adjustment for multiple comparisons
☐	☒	A full description of the statistical parameters including central tendency (e.g. means) or other basic estimates (e.g. regression coefficient) AND variation (e.g. standard deviation) or associated estimates of uncertainty (e.g. confidence intervals)
☐	☒	For null hypothesis testing, the test statistic (e.g. *F*, *t*, *r*) with confidence intervals, effect sizes, degrees of freedom and *P* value noted<br>*Give P values as exact values whenever suitable.*
☒	☐	For Bayesian analysis, information on the choice of priors and Markov chain Monte Carlo settings
☒	☐	For hierarchical and complex designs, identification of the appropriate level for tests and full reporting of outcomes
☐	☒	Estimates of effect sizes (e.g. Cohen's *d*, Pearson's *r*), indicating how they were calculated

Our web collection on statistics for biologists contains articles on many of the points above.

Software and code

Policy information about availability of computer code

Data collection

No software was used for data collection.

Data analysis

Softwares used in this study are publically available and stated in the Methods section where applicable.
-mIF: inForm 2.4.8 Analysis software
-gEOmX: GeoMx DSP analysis suite version 2.2.0.111
- WES analysis: BWA-MEM version 0.7.17 (GRCh37), Picard v2.18.11, GATK v4.1.3.0
-Tumor content: Sequenza 3.0.0
-Mutation calling: HaplotypeCaller (GATK v4.1.3.0); MuTect v1.1.7; Mutect v2 (GATK v4.1.3.0); and VarScan 2 (v2.4.3), SAMtools v1.8
-HLA typing: Assign TruSight software v2.1
-RNA sequencing analyses and postprocessing: RNA-Star v2.7.3a, GTEx v7, limma v.3.36.5 (Bioconductor), GENCODE annotation 32.
-MS analyses: Comet 2019015, NewAnce v1.7.4 (see below), Spectronaut v14.6.2. Spectronaut v16.3
-HLA-peptide binding prediction: MixMHCpred v2.1, MixMHC2pred v1.2
-TE analyses: limma v3.36.5
-Smoking Signatures: Palimpsest (v2.0.0)
-Tree construction: LICHEe v1.0
-NewAnce v1.7.1 : An executable jar file of NewAnce has been deposited to PRIDE with the dataset identifier PXD013649. The NewAnce code is available on the following GitHub link https://github.com/bassanilab/NewAnce.git.
-General: R 4.0.2 and Python 3.6, STRING-db v11, GTEx v7, RStudio 1.3.959
R packages:
ggforce_0.4.1, vcd_1.4-11, ggsignif_0.6.4, corrplot_0.92, biomaRt_2.46.3, ggh4x_0.2.2, edgeR_3.30.3, limma_3.44.3, mixOmics_6.12.2
lattice_0.20-45, MASS_7.3-56, ggpubr_0.4.0, forcats_1.0.0, stringr_1.4.1

purrr_0.3.4, readr_2.1.2, tibble_3.1.8, tidyverse_1.3.2, topGO_2.40.0, SparseM_1.81, GO.db_3.11.4, AnnotationDbi_1.52.0, IRanges_2.22.2 S4Vectors_0.26.1, Biobase_2.48.0, graph_1.66.0, BiocGenerics_0.36.1, RColorBrewer_1.1-3, jsonlite_1.8.0, rjson_0.2.21, gplots_3.1.3, cowplot_1.1.1, tidyr_1.2.0, ggplot2_3.3.6, dplyr_1.0.10,ggh4x_0.2.2

For manuscripts utilizing custom algorithms or software that are central to the research but not yet described in published literature, software must be made available to editors and reviewers. We strongly encourage code deposition in a community repository (e.g. GitHub). See the Nature Portfolio guidelines for submitting code & software for further information.

Data

Policy information about availability of data

All manuscripts must include a data availability statement. This statement should provide the following information, where applicable:

- Accession codes, unique identifiers, or web links for publicly available datasets
- A description of any restrictions on data availability
- For clinical datasets or third party data, please ensure that the statement adheres to our policy

The datasets generated and analyzed during this study are available in the European Genome phenome Archive (EGA), and can be accessed with the ID EGAS00001006298. Mass spectrometry data and Spectronaut parameters are available via ProteomeXchange with identifier PXD034772.

The TRACERx NSCLC WES and RNAseq data files were downloaded from the EGA archive (EGAD00001004591 and EGAD00001003206). Gartner et al. WES and RNAseq data were downloaded from dbGap accession number phs001003v1.p1. Source data have been provided as Source Data files. All other data supporting the findings of this study are available from the corresponding author on reasonable request.

Databases can be accessed through:
ProteinAtlas: www.proteinatlas.org/about/download
TCGA: www.cancer.gov/about-nci/organization/ccg/research/structural-genomics/tcga/using-tcga/citing-tcga
NCBI (Virus): support.nlm.nih.gov/knowledgebase/article/KA-03391/en-us
GTEx: www.gtexportal.org/home/datasets
ENSEMBL: https://www.ensembl.org/info/about/publications.html
COSMIC: cancer.sanger.ac.uk/cosmic/license
SNPeff: pcingola.github.io/SnpEff/
GENCODE: www.gencodegenes.org/human/release_32lift37.html
Refseq: www.ncbi.nlm.nih.gov/assembly/GCF_000001405.13/
nuORFdb: www.nature.com/articles/s41587-021-01021-3#MOESM4
GenBank CDS translations: ncbi.nlm.nih.gov/genbank
PDB: rcsb.org
Uniprot: https://www.uniprot.org/
PIR: proteininformationresource.org
PRF: prf.or.jp

The source data underlying the Figures and Supplementary Figures, where applicable, are provided as a Source Data file

Human research participants

Policy information about studies involving human research participants and Sex and Gender in Research.

Reporting on sex and gender	Male/female infomration was collected based on informed concent. In this small cohort separate analysis based on sex and gender was not performed. No correlations with sex or gender were performed or analyzed.
Population characteristics	This information is available in Supplemental Table 1.<br>Sample_name Cancer_Type Sex Grade<br>C3N-02672 LUAD female G3<br>C3N-02671 LUAD female G2<br>C3N-02287 LUAD male G3<br>C3N-02288 LUSC female G2<br>C3N-02289 LUSC male G2<br>C3N-02290 LUAD male G2<br>C3N-03023 LCNEC female G3<br>C3N-03421 LUAD female G2
Recruitment	Tissues were collected and biobanked. We selected all available tissues from these patients. The sample material was in all cases large enough to conduct immunopeptidomics, DNA and RNA extraction and FFPE staining. The selection of samples should not have any impact on the results obtained.
Ethics oversight	Informed consent of the participants was obtained following requirements of the institutional review boare (Ethics Commission CHUV, Bioethics Committee, Poznan University of Medical Sciences, Poznan, Poland).

Note that full information on the approval of the study protocol must also be provided in the manuscript.

Field-specific reporting

Please select the one below that is the best fit for your research. If you are not sure, read the appropriate sections before making your selection.

☒ Life sciences ☐ Behavioural & social sciences ☐ Ecological, evolutionary & environmental sciences

For a reference copy of the document with all sections, see nature.com/documents/nr-reporting-summary-flat.pdf

Life sciences study design

All studies must disclose on these points even when the disclosure is negative.

Sample size	We surveyed 8 Patients and in total 61 regions. Samples were collected and included in this exploratory study only Tbased on their availability.
Data exclusions	No patients or macro-regions of the 8 Lung cohort patients were excluded throughout the analysis. From the TRACERx cohort, we excluded from the analysis samples CRUK0079-R3 due to RNAseq pipeline errors, CRUK0004 because no synonynmous mutations were found, and CRUK0012 because only 3 alleles were available for predictions and no synonymous mutations predicted to be binders to the patient's HLA were found. In the RosenbergNCI dataset, we excluded patients 1913, 2098, 2224 and 3309 were as for those only positive n mers were included in the dataset.
Replication	Technical replicates were performed on the MS measurment of DDA or DIA samples. No oarticular measures were set to test reproducibility of methods.
Randomization	Data was not randomized.
Blinding	Data collection and analysis were not performed blind to the conditions of the experiments.

Reporting for specific materials, systems and methods

We require information from authors about some types of materials, experimental systems and methods used in many studies. Here, indicate whether each material, system or method listed is relevant to your study. If you are not sure if a list item applies to your research, read the appropriate section before selecting a response.

Materials & experimental systems

n/a	Involved in the study	
☐	☒	Antibodies
☐	☒	Eukaryotic cell lines
☒	☐	Palaeontology and archaeology
☒	☐	Animals and other organisms
☒	☐	Clinical data
☒	☐	Dual use research of concern

Methods

n/a	Involved in the study	
☒	☐	ChIP-seq
☒	☐	Flow cytometry
☒	☐	MRI-based neuroimaging

Antibodies

Antibodies used	Multiplex staining consisted in multiple rounds of staining. Each round of multiplex staining included: non-specific sites blocking (DISCOVERY Goat Ig Block (#760-6008) and DISCOVERY Inhibitor (#760-4840), Roche Diagnostics), primary antibody incubation, secondary HRP-labeled antibody incubation for 16 minutes (DISCOVERY OmniMap anti-Rb HRP (#760-4311, Roche Diagnostics) or DISCOVERY OmniMap anti-Ms HRP (#760-4310, Roche Diagnostics)), OPALTM reactive fluorophore detection (Akoya Biosciences, Marlborough, MS, USA) that covalently label the primary epitope (incubation time of 12 minutes) followed by antibodies heat denaturation (100°C for 8 minutes).<br>Sequence of antibodies fFor the first panel:, the following sequence of antibodies was used in the multiplex staining with the associated OPAL: mouse monoclonal anti-human PD1 antibody (clone NAT105, Biocare, # ACI3137C, 1hour, RT), OPAL570 (Akoya Biosciences, # FP1488001KT); rabbit polyclonal anti-CD3 antibody (0.4 g/l, Dako, # A0452, 32 minutes, 37°C), OPAL480 (Akoya Biosciences, # FP1500001KT); mouse monoclonal anti-GranzymeB antibody, Clone GrB-7, Monosan, # MON7029-1, 1hour, RT), OPAL620 (Akoya Biosciences, # FP1495001KT); rabbit monoclonal anti-Ki67 antibody (1 µg/ml, Clone SP6, Cellmarque, # 275R-16, 1hour, 37°C), OPAL520 (Akoya Biosciences, # FP1487001KT); mouse monoclonal anti-Cytokeratin antibody (1 µg/ml, Clone AE1/AE3, Dako, # M3515, 1hour, RT), OPAL690 (Akoya Biosciences, # FP1497001KT); rabbit monoclonal anti-CD8 antibody (76.9 µg/ml, clone SP16, Cellmarque, # 108R-16-RUO, 1hour, 37°C), OPAL780 (Akoya Biosciences, # FP1501001KT). Sequence of antibodies fFor the second panel, the following sequence of antibodies was used with the associated OPAL: rabbit polyclonal anti-CD3 antibody, OPAL570; rabbit monoclonal anti-human FoxP3 antibody (clone SP97, Spring, # M3974, 1hour, 37°C), OPAL520; rabbit polyclonal anti-CD20 antibody (126 mg/l, Dako, # M0755, 1hour, 37°C), OPAL620 ; mouse monoclonal anti-HLA-DR antibody (Clone TAL-1B5, Dako, # M0746, 1hour, 37°C), OPAL480; mouse monoclonal anti-Cytokeratin antibody, OPAL690; rabbit monoclonal anti-CD8

March 2021

antibody, OPAL780. Nuclei were visualized by a final incubation with Spectral DAPI (1/10, # FP1490, Akoya Biosciences) for 12 minutes. Slides were mounted with Fluorescence Mounting Medium (Agilent Technoligies, # S302380-2) and coverslipped.

Anti HLA-I antibody : from hybridoma “HB-95”
Company name : ATCC
Catalog number : HB-95
Lot number: 7001294
Clone name: W6/32
Antigenic determinant: HLA-A, B, C
Isotype: IgG2a
Host: mouse
Cell type: Hybridoma: B lymphocyte
Clonality: monoclonal
Amount used: 1mg per 1ml of Protein A beads.

Anti HLA-II antibody : from hybridoma “HB-145”
Company name : ATCC
Catalog number : HB-145
Lot number:59681660
Clone name: iva12
Amount used: 1mg per 1ml of Protein A beads.

Validation

Validation by vendor following ATCC guidelines. Certificate of Analysis can be found here:https://www.lgcstandards-atcc.org/Products/All/HB-95.aspx?geo_country=ch#documentation and https://www.lgcstandards-atcc.org/Products/All/HB-145.aspx?geo_country=ch#documentation
Additionally, anti-HLA-I and -II antibodies were validated directly in our laboratory, through the use of these antibodies for immuno-affinity purification of HLA-I and -II peptides from cell lines and tissue samples. These peptides were measured by mass spectrometry, and their characteristics fit that of HLA-I and -II peptides, respectively.

Eukaryotic cell lines

Policy information about cell lines and Sex and Gender in Research

Cell line source(s)

nti HLA-I antibody : from hybridoma “HB-95”
Company name : ATCC

Anti HLA-II antibody : from hybridoma “HB-145”
Company name : ATCC

Authentication

Validation by vendor following ATCC guidelines. Certificate of Analysis can be found here:https://www.lgcstandards-atcc.org/Products/All/HB-95.aspx?geo_country=ch#documentation and https://www.lgcstandards-atcc.org/Products/All/HB-145.aspx?
geo_country=ch#documentation

Mycoplasma contamination

All cell lines were tested negative for mycoplasma.

Commonly misidentified lines
(See ICLAC register)

Not applicable.
